# Tetracyclines in the Modern Era: Global Consumption, Antimicrobial Resistance, Environmental Occurrence, and Degradation Techniques

**DOI:** 10.3390/antibiotics14121183

**Published:** 2025-11-21

**Authors:** Yuliya Semenova, Larissa Makalkina, Natalya Glushkova, Abduzhappar Gaipov

**Affiliations:** 1 Department of Surgery, Nazarbayev University School of Medicine, Astana 010000, Kazakhstan; 2 Department of Clinical Pharmacology, Astana Medical University, Astana 010000, Kazakhstan; 3 Department of Epidemiology, Biostatistics and Evidence-Based Medicine, Al-Farabi Kazakh National University, Almaty 050040, Kazakhstan; glushkova.natalya@kaznu.kz; 4 Department of Clinical Medicine, Nazarbayev University School of Medicine, Astana 010000, Kazakhstan; abduzhappar.gaipov@nu.edu.kz

**Keywords:** tetracyclines, antimicrobial resistance, environment, antimicrobial stewardship, one health, degradation

## Abstract

Tetracyclines are among the oldest classes of antibiotics, with broad activity against Gram-positive and Gram-negative bacteria, as well as *Chlamydia*, *Legionella*, *Rickettsia*, and *Mycoplasma* species. Widely used in human and veterinary medicine, agriculture, and aquaculture, they represent approximately 10–12% of the global antimicrobial market. Extensive use has driven the emergence and spread of antimicrobial resistance, posing ecological and public health risks. However, the full extent of these risks remains unclear due to limited data on tetracycline consumption, environmental occurrence, and resistance patterns across sectors. Recent One Health-oriented strategies have promoted the rational use of tetracyclines in medicine, veterinary practice, and agriculture. To reduce environmental accumulation, various degradation and remediation techniques have been developed, though most remain restricted to laboratory or engineered settings. This narrative review provides a comprehensive overview of global tetracycline consumption; environmental occurrence; distribution and concentration levels; resistance mechanisms and prevalence; and mitigation strategies, including antimicrobial stewardship and degradation approaches. Understanding these aspects is essential for developing evidence-based interventions to minimize the environmental and public health impacts of tetracycline use.

## 1. Introduction

Tetracyclines are among the oldest groups of antibiotics, characterized by a broad spectrum of activity effective against both Gram-positive and Gram-negative bacteria, as well as *Chlamydia*, *Legionella*, *Rickettsia*, and *Mycoplasma* species [1]. They have wide-ranging applications in human and veterinary medicine, agriculture, and aquaculture, accounting for approximately 10–12% of the global antimicrobial market [2]. The use of tetracyclines in human medicine is less common than in veterinary practice due to the availability of newer antibiotic classes. However, tetracyclines remain extensively used in veterinary medicine for the treatment of zoonotic infections and as growth promoters, as they are inexpensive, well absorbed, and generally associated with few adverse effects [3]. The widespread global use of tetracyclines has led to their pervasive distribution in the environment, resulting in several negative ecological and public health consequences.

The ecological consequences of tetracycline occurrence in the environment include disruption of terrestrial and aquatic microbiota, which affects organic matter decomposition and plant growth processes [4]. Tetracyclines can also exert toxic effects on non-target organisms such as algae, invertebrates, and fish, leading to growth inhibition and reproductive toxicity [5]. Moreover, tetracyclines are capable of bioaccumulation, potentially resulting in biomagnification through the food chain and chronic exposure in higher organisms, including humans [6]. In addition to their toxic effects, tetracyclines contribute to the development and dissemination of antimicrobial resistance (AMR), posing significant ecological and public health risks.

AMR refers to the ability of microorganisms to survive exposure to antimicrobial agents that would normally inhibit their growth or cause cell death [7]. The overuse and misuse of antimicrobial agents are major drivers of AMR development [8]. In particular, the extensive application of tetracyclines in human and veterinary medicine, agriculture, and aquaculture results in the persistence of tetracycline residues in soil, surface water, and sediments. These residues exert continuous selective pressure, promoting the maintenance and dissemination of tetracycline-resistant microorganisms [9]. Consequently, the environment acts as both a reservoir and a conduit for AMR spread, facilitating the potential reintroduction of resistant bacteria into human and animal populations through water, food, and direct contact pathways [10]. To address the growing problem of tetracycline resistance, a range of strategies has been proposed. Antimicrobial stewardship (AMS) practices promote the rational use of tetracyclines in human and animal health sectors [11], while a variety of degradation techniques have been developed to remove tetracyclines already present in the environment [12].

Although numerous studies have examined individual aspects of tetracycline use, environmental fate, or resistance, most existing reviews address these topics in isolation. There remains a lack of an updated, integrated synthesis that simultaneously examines global consumption of tetracyclines across major sectors; their persistence in terrestrial and aquatic environments; the mechanisms of resistance; and the full spectrum of mitigation strategies, including AMS interventions and emerging degradation approaches, within a unified One Health framework [13]. The innovation of this narrative review lies in its integrative scope. By synthesizing evidence from human and veterinary medicine, agriculture, aquaculture, environmental pollution, and public health, it provides a state-of-the-art, cross-sectoral overview. Through this holistic perspective, the review aims to support the development of evidence-based interventions to mitigate the environmental and public health impacts associated with tetracycline use.

## 2. Tetracycline Class of Antibiotics

Tetracyclines are a class of antibiotics originally derived from bacteria of the *Streptomyces* genus. Chlortetracycline, oxytetracycline, tetracycline, and demeclocycline constitute the first generation of tetracyclines. The first antibiotic in this class, chlortetracycline, was isolated from *Streptomyces aureofaciens* in 1945 by Benjamin Minge Duggar at Lederle Laboratories. Oxytetracycline was the second to be discovered, isolated from *Streptomyces rimosus* in 1949 by a research team at Charles Pfizer & Co., including John and Alexander Finlay and Frank E. Hegeman. Tetracycline was subsequently developed as a semisynthetic hydrogenated derivative of chlortetracycline by Lloyd Conover at Pfizer and approved for clinical use in 1953. Demeclocycline was later isolated in 1957 by Jerry Robert Daniel McCormick and colleagues from a mutant strain of *Streptomyces aureofaciens* [14]. The second-generation tetracyclines—such as minocycline, doxycycline, lymecycline, rolitetracycline, and methacycline—emerged during the 1960s and 1970s and were developed to improve pharmacokinetic profiles and spectrum of activity [15]. The third generation, known as glycylcyclines (e.g., tigecycline, omadacycline, sarecycline, and eravacycline), consists of fully synthetic analogues developed in the 2000s and 2010s to overcome increasing bacterial resistance [16]. Despite structural modifications across generations, all tetracyclines share a characteristic core structure.

### 2.1. Chemical Structure and Mechanism of Action

**Figure 1:**
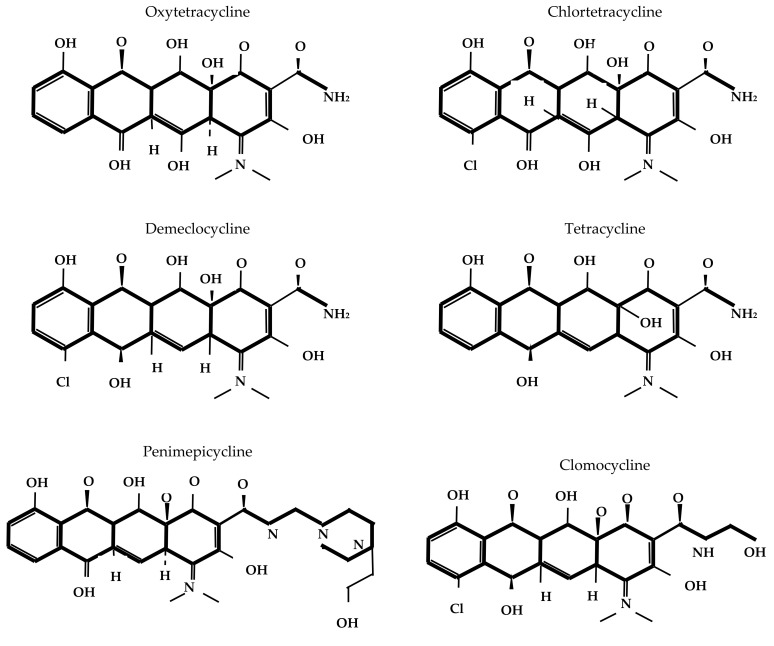

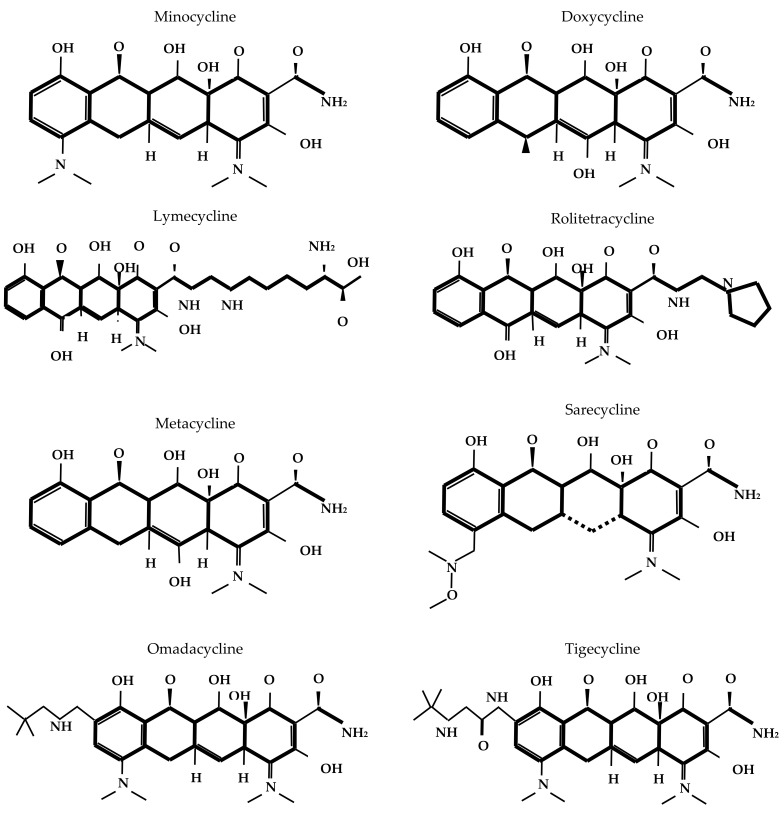
The structure of tetracyclines.

The core structure of tetracycline molecules consists of four linearly fused six-membered rings (conventionally labeled A, B, C, and D), forming the characteristic naphthacene ring system. In this scaffold, ring A typically bears a dimethylamino group at position C4; rings B and C contain key keto-enol tautomeric functionalities; and ring D carries hydroxyl or other substituents, depending on the specific derivative. Structural variations at positions C5, C6, C7, and C9 contribute to the pharmacokinetic, antimicrobial, and resistance profiles of different tetracycline compounds [17]. Figure 1 depicts the chemical structure of different tetracyclines.

Tetracyclines possess bacteriostatic activity, and their mode of antibacterial action involves the inhibition of protein synthesis at the bacterial ribosome. Specifically, tetracyclines bind reversibly to the 16S rRNA component of the 30S ribosomal subunit in susceptible bacteria, distorting the A-site and blocking the attachment of aminoacyl-tRNA. This interference disrupts the codon–anticodon pairing and blocks the addition of new amino acids to the elongating polypeptide chain. As a result, protein synthesis is arrested at the elongation stage, leading to a bacteriostatic effect [18]. However, it was demonstrated that tetracyclines may exhibit bactericidal properties at high concentrations, potentially due to their ability to disrupt the functional integrity of cytoplasmic membrane [19]. The binding of tetracyclines to the bacterial ribosome is reversible, which accounts for their primarily bacteriostatic nature and relatively modest post-antibiotic effect [17].

Intracellular accumulation of tetracyclines is essential for their antibacterial efficacy and underlies their selective toxicity. Tetracyclines typically enter the periplasm of bacteria via energy-independent diffusion of a tetracycline–Mg^2+^ complex through outer membrane porins [20]. Subsequently, the Donnan potential aids dissociation of the tetracycline–Mg^2+^ complex, resulting in an uncharged, lipophilic tetracycline molecule that can diffuse across the cytoplasmic membrane [21]. Moreover, energy-dependent uptake occurs via the proton motive force and ATP hydrolysis, contributing to active transport across the cytoplasmic membrane [17]. Inside the cell, tetracyclines undergo repeated chelation with Mg^2+^ ions, stabilizing their interaction with the ribosomal target [16]. It has to be noted that tetracycline molecules rely partially on bacterial-specific transport systems that are absent or significantly less active in mammalian cells, which explains their selective inhibition of bacterial protein synthesis with relatively low cytotoxicity in host tissues [22].

Beyond their antibacterial effects, certain tetracyclines, particularly doxycycline and minocycline, demonstrate non-antibiotic properties, such as anti-inflammatory activity. These effects have expanded their therapeutic applications to include conditions like acne vulgaris, rosacea, periodontitis, rheumatoid arthritis, and inflammatory bowel disease [23]. Moreover, tetracyclines such as doxycycline, minocycline, and tigecycline have shown anticancer potential through mechanisms involving mitochondrial dysfunction, inhibition of matrix metalloproteinases, and apoptosis induction [24]. In addition to their antibacterial and anti-inflammatory roles, doxycycline and minocycline have also been investigated for their potential therapeutic effects in neuropsychiatric conditions, including major depressive disorder [25] and neurodegenerative diseases such as Alzheimer’s disease [26].

Tetracycline-class antibiotics are classified based on their elimination half-life (EHL) into short-acting, intermediate-acting, and long-acting agents. Short-acting tetracyclines, such as tetracycline, oxytetracycline, and chlortetracycline, have an EHL of approximately 6 to 8 h. Intermediate-acting agents include demethylchlortetracycline, lymecycline, rolitetracycline, clomocycline, penimepicycline, and methacycline, with an EHL of 12 to 15 h. In contrast, long-acting tetracyclines—such as doxycycline, minocycline, sarecycline, eravacycline, omadacycline, and tigecycline—have an EHL exceeding 16 h [17]. The EHL of tetracyclines depends on their pharmacokinetic characteristics, including absorption, distribution, metabolism, and excretion pathways. Short- and intermediate-acting tetracyclines are primarily excreted via the kidneys, whereas long-acting tetracyclines are predominantly excreted through the gastrointestinal tract [14].

### 2.2. Spectrum of Antibacterial Activity and Indications for Use

Tetracyclines are broad-spectrum antibiotics, effective against a wide range of aerobic and anaerobic, Gram-positive and Gram-negative bacteria (Table 1). In addition to the listed bacteria, new and rare pathogens occasionally found to be sensitive to tetracyclines [27].

Beyond bacterial pathogens, tetracyclines are also effective in infections caused by some protozoa and parasites, like *Entamoeba histolytica* and *Plasmodium* spp., and in bacterial infections closely related to treponemal diseases, such as yaws caused by *Treponema pallidum* subspecies *pertenue* [28].

Tetracyclines possess a broad antimicrobial spectrum and exhibit extensive tissue distribution, which makes them effective in treating both systemic and localized infections caused by susceptible organisms. Although tetracyclines penetrate nearly all body tissues, the highest concentrations are typically observed in the bile, liver, spleen, kidneys, lungs, and bone [29]. Notably, doxycycline and minocycline have been shown to cross the blood–brain barrier, allowing for potential use in central nervous system infections [17].

In veterinary medicine, tetracyclines are used not only for therapeutic purposes but also as growth promoters, often added to animal feed at subtherapeutic levels. This practice enhances feed efficiency and weight gain, primarily by suppressing subclinical infections and reducing immune stress in intensive breeding environments. It may also improve intestinal health and nutrient utilization indirectly through modulation of gut microbiota composition. However, the use of tetracyclines in animals for growth promotion is increasingly restricted due to concerns over associated resistance [30]. Additionally, tetracyclines are employed in aquaculture to manage infections in commercially farmed fish, in agriculture to treat bacterial diseases in trees and plants, and in insect farming to prevent and treat infections in bees and other economically important insects [17].

Despite their initially broad spectrum of antimicrobial activity, the effectiveness of tetracyclines in the prevention and treatment of infections has been significantly compromised by the emergence and spread of resistance. This resistance is largely attributed to the widespread use of tetracyclines in both human and veterinary medicine. Understanding the patterns of tetracycline consumption is essential for guiding AMS strategies.

## 3. Global Consumption of Tetracyclines

The consumption of tetracyclines in the human health sector is substantially lower than in the animal health sector. In human medicine, tetracyclines are not typically used as first-line treatments for many common infections, which limits their overall volume of use. In contrast, tetracyclines are widely used in veterinary medicine due to their broad-spectrum activity, low cost, favorable safety profile, and historical application for non-therapeutic purposes such as growth promotion [17]. To monitor these patterns, several international and national agencies collect and publish data on antibiotic consumption in both human and veterinary sectors, including the WHO, the European Medicines Agency (EMA), the Centers for Disease Control and Prevention (CDC), the U.S. Food and Drug Administration (FDA), and the WOAH.

### 3.1. Consumption of Tetacyclines in Human Health Sector

To enable cross-regional, cross-national, and temporal comparisons of antimicrobial consumption, the WHO developed a standardized methodology that expresses medicine use as defined daily doses per 1000 individuals per day. According to this methodology, all antibiotic agents are assigned a defined daily dose (DDD), which represents the assumed average maintenance dose per day for a drug used for its main indication in adults [31]. This metric allows for harmonized reporting and comparison of antibiotic use between different countries and healthcare systems, regardless of population size or prescribing practices [32].

At the global level, the European region is the most extensively studied in terms of antibiotic consumption based on the DDD methodology. In the European Union (EU), data are collected and analyzed by the European Centre for Disease Prevention and Control. According to the latest available report (2023), the highest tetracycline consumption was reported in Iceland (4.1 DDD), followed by Ireland (3.96 DDD) and France (2.8 DDD). In contrast, Austria (0.4 DDD), Slovenia (0.7 DDD), and Italy (0.6 DDD) recorded the lowest levels [33]. Among non-EU European countries, the WHO Regional Office for Europe monitors tetracycline consumption. The highest reported consumption in this group was observed in Albania (2.6 DDD), followed by Serbia and Montenegro (1.8 DDD each) [34].

In Asia, the WHO Regional Office for the Western Pacific monitors and reports antibiotic consumption data. Within this region, the Lao People’s Democratic Republic had the highest tetracycline consumption at 2.9 DDD, followed by Hong Kong (1.8 DDD) and Mongolia (1.6 DDD) [35]. In China, the latest available data are limited to a single province, Shandong, which reported 0.13 DDD [36]. In the Latin America and Caribbean region, Barbados reported the highest tetracycline consumption (4.2 DDD), while Paraguay had the lowest (0.02 DDD) [37]. In Canada, tetracycline consumption was estimated at 1.2 DDD [38].

**Figure 2:**
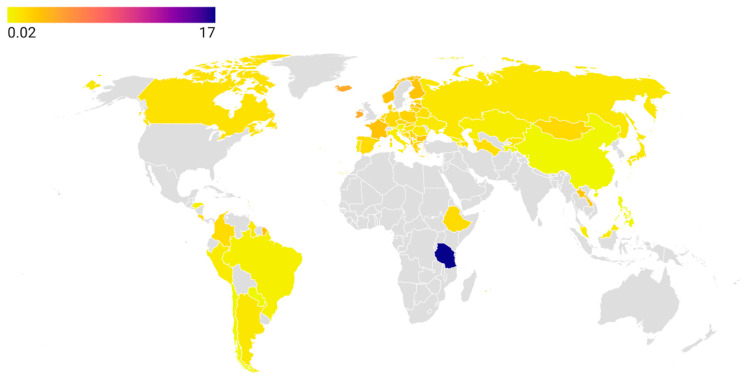
Consumption of tetracyclines expressed as defined daily doses per 1000 inhabitants per day across 65 countries and territories over the past five years. The grey areas represent regions with no available reports.

Although the African region remains the most understudied in terms of tetracycline consumption, it includes the country with the highest reported global use: Tanzania (17.0 DDD in 2019), which needs to be investigated further [39]. In contrast, Ethiopia reported significantly lower usage at 1.47 DDD [40]. Figure 2 displays a choropleth map of tetracycline consumption (expressed in DDD) across 65 countries and territories over the past five years, while Table S1 provides the corresponding numerical data and references to the data sources used.

Another approach to assessing tetracycline consumption in human medicine is by examining their proportion relative to total antibiotic use. According to the Global Antimicrobial Resistance and Use Surveillance System report published by the WHO in 2022, tetracyclines were the fifth most consumed class of antibiotics globally, following penicillins, macrolides, penicillin–beta-lactamase inhibitor combinations, and fluoroquinolones. The median global proportion of tetracycline use was 7.7%. Among tetracyclines, doxycycline was the most commonly used and ranked as the most prescribed antibiotic in two high-income countries. Its median proportional use among oral antibiotics was 7.4% [41].

### 3.2. Consumption of Tetracyclines in Veterinary Medicine

Tetracycline consumption in veterinary medicine far exceeds that in human medicine. For instance, in the EU in 2021, the population-weighted mean consumption was 1.9 mg/kg of estimated biomass in humans compared to 23.6 mg/kg in food-producing animals [42]. This significantly higher use of tetracyclines in the veterinary sector warrants further investigation.

In global veterinary medicine, tetracyclines represent the most widely consumed class of antimicrobials, with an estimated 33,305 tonnes used in 2020 [43]. Since 2015, the WOAH has collected data on antimicrobial use in animal medicine through the global database on animal antimicrobial use (ANIMUSE). National veterinary authorities from WOAH member countries submit annual data on antimicrobial consumption and choose whether to share it publicly or privately. Among 85 participating countries, 53 have opted for public data sharing, and the most recent data are available for 2023 [44].

**Figure 3:**
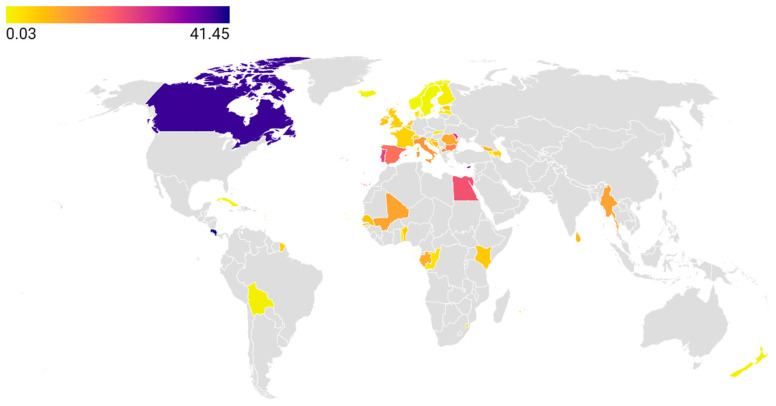
Tetracycline consumption, expressed in milligrams per kilogram of estimated animal biomass, across 53 countries and territories reporting to the World Organisation for Animal Health over the past five years. The grey areas represent regions with no available reports.

According to the ANIMUSE database, tetracycline consumption rates are most comprehensively studied in the European region. As of 2023, Cyprus reported the highest level of consumption at 33.1 mg/kg, followed by Moldova (26.6 mg/kg) and Bulgaria (15.0 mg/kg). The lowest rates were observed in Norway (0.03 mg/kg), Sweden (0.45 mg/kg), and Iceland (0.57 mg/kg). There is a paucity of data from Asian countries, as only a few have agreed to publicly share their consumption rates. Reported values include Myanmar (10.8 mg/kg) and Sri Lanka (7.57 mg/kg). In Africa, the highest consumption rates were observed in Egypt (21.57 mg/kg), followed by Mali (10.64 mg/kg) and Gabon (9.33 mg/kg), while the lowest was reported in Cape Verde (0.16 mg/kg). In Latin America and the Caribbean, the highest rate was reported in Costa Rica (41.44 mg/kg) and the lowest in Cuba (0.41 mg/kg). Notably, Canada reported the highest tetracycline consumption rate globally in the animal sector, at 39.2 mg/kg [44] (Figure 3, Table S2).

It is plausible that countries opting to report their antimicrobial use data to the WOAH privately may have higher tetracycline consumption rates. A willingness to share data publicly may reflect national authorities’ confidence in maintaining relatively modest levels of antimicrobial use. Notably, tetracycline consumption data are not publicly available for several of the world’s leading producers of farmed animals, including India, China, the United States, and Brazil [45]. However, some publicly available sources provide estimates of tetracycline use in these countries. For example, the FDA reported 3.92 tonnes of tetracyclines sold for livestock in 2021, representing 65% of all medically important antimicrobials used in animals that year, with the majority administered via feed (3.28 tonnes) [46]. In China, tetracycline consumption in food-producing animals was estimated at 10,002.73 tonnes in 2020 [47]. Although Brazil does not publicly report antimicrobial consumption by class, it has been identified as one of the top five global consumers of veterinary antimicrobials [43]. Despite recent downward trends in tetracycline use in both the United States and China [46,47], these countries remain among the highest global consumers.

According to the latest annual report on antimicrobial agents intended for use in animals issued by the WOAH, tetracyclines were the most consumed class of antimicrobials globally, accounting for 26.3% of total use, followed by penicillins at 17.75%. Although previous modelling studн projected an increase in tetracycline consumption [43], recent WOAH data show a declining trend in tetracyclines’ use and a concurrent rise in the share of penicillins. This decline is not observed in Europe but is more pronounced in the Americas, Asia, and the Pacific. Thirty-three WOAH member countries provided a breakdown of antimicrobial classes used for growth promotion purposes. As of 2023, oxytetracycline was used for growth promotion in eight countries, chlortetracycline in five countries, and tetracycline in one country. The global mean consumption of tetracyclines in terrestrial food-producing animals was estimated at 24.26 mg per kg of animal biomass [47].

The latest WOAH report also provides insights into tetracycline use in aquaculture. As of 2022, tetracyclines were the second most consumed class of antimicrobials in aquatic food-producing animals (26.18%), following amphenicols (27.41%). However, oxytetracycline was the single most used antimicrobial agent, accounting for 32% of total use in this sector. Doxycycline was also used, though less frequently, representing 5% of total use. The global mean consumption of tetracyclines in aquatic food-producing animals was estimated at 6.43 mg per kg of animal biomass [48].

Although the share of tetracyclines is declining in terrestrial food-producing animals and they are not the most commonly used class in aquaculture, these agents continue to play a major role in veterinary medicine. Continued monitoring of tetracycline use in both sectors remains essential for global AMS.

### 3.3. Consumption of Tetracyclines in Commercial Plant Agriculture

Oxytetracycline is the second most commonly used antibiotic in plant agriculture, following streptomycin. In the Americas, it is applied in fruit orchards producing citrus, peaches, apricots, apples, pears, and in tropical plantations growing bananas and plantains [49]. In Southeast Asia and China, oxytetracycline is used in rice cultivation and vegetable farming, particularly for crops such as tomatoes, cabbage, and potatoes. In parts of Europe, oxytetracycline has also been used in commercially grown berry crops, including strawberry [50].

Unlike in human and veterinary medicine, there is no international organization that systematically monitors tetracycline use in commercial plant agriculture on a global scale. The FAO addresses AMR in plant production systems, focusing on transmission pathways such as manure, wastewater, and pesticide applications, including the use of antibiotics as bactericides. The InFARM system (International FAO Antimicrobial Resistance Monitoring system), launched by FAO in 2022, aims to strengthen national capacities for AMR surveillance across food and agriculture sectors [51]. However, plant agriculture remains the least represented sector in this system, and country participation in reporting plant-specific antimicrobial use is voluntary and currently limited.

The United States is arguably the only country that systematically collects and reports data on antibiotic use in plant agriculture. The National Agricultural Statistics Service of the U.S. Department of Agriculture is responsible for this data collection and regularly publishes usage reports by antibiotic class. As of 2023, approximately 18 tonnes of oxytetracycline were applied to apple, peach, orange, and pear orchards. This was lower than the 25.5 tonnes of streptomycin used on the same fruit crops in the same year [52]. A comparison with historical data shows a gradual increase in oxytetracycline use, from less than 10 tonnes in 1991 [49]. Although it is also applied to non-orchard crops, these quantities are presumed to be minimal and are not systematically reported [52].

In general, tetracycline use in crop agriculture in the United States remains low and is estimated to represent only a small fraction of the amount used in veterinary medicine. In contrast, consumption in lower- and middle-income countries (LMICs) is believed to be considerably higher. In LMICs with large-scale crop production, tetracycline use in plant agriculture may even exceed that in human or veterinary medicine [53]. For example, in Costa Rica, the estimated amount of oxytetracycline used in crops is reported to be 200 to 700 times greater than that used in human medicine [54]. Southeast Asia is thought to be the highest-consuming region globally, with approximately 7 tonnes of tetracyclines used annually for rice cultivation alone [53]. A study from the Indian Center for Science and Environment showed that farmers apply streptocycline (a combination of streptomycin and tetracycline in a 9:1 ratio) to a wide range of crops as frequently as once every two weeks [55], suggesting that actual tetracycline use in the region may be even higher than estimated.

Outside of the United States, antibiotic use in crops remains poorly monitored. Nonetheless, the FAO estimates that plant agriculture accounts for approximately 0.26–0.5% of total global antibiotic consumption. An added complexity is the widespread use of manure from food-producing animals as fertilizer in crop fields. In countries where tetracyclines are heavily used in animal agriculture, this practice may lead to significant residual contamination of crops. Consequently, the actual concentrations of tetracyclines in plant products may exceed estimated levels [56]. Therefore, the contribution of plant agriculture to the dissemination of tetracycline resistance should not be underestimated.

## 4. Tetracyclines and the Environment

First-generation tetracyclines are primarily excreted via the urine, whereas second- and third-generation tetracyclines are mainly excreted through bile, with 50–80% of the drug excreted unchanged [57]. Once released into the environment, tetracyclines exhibit considerable stability due to their resistance to oxidation. Although tetracyclines may become unstable under extreme pH conditions, transforming into epi- and anhydro-forms, the resulting compounds are characterized by low volatility and high environmental persistence [58]. Moreover, tetracyclines readily form strong complexes with metal ions, such as Mg^2+^ and Ca^2+^, further reducing their bioavailability for degradation. As a result, these compounds tend to accumulate in both terrestrial and aquatic environments [4].

### 4.1. Environmental Cycle of Tetracyclines

After their use in human and veterinary medicine, tetracyclines are excreted and enter wastewater treatment plants (WWTPs). However, modern WWTPs are often not equipped to effectively remove antibiotics, including tetracyclines, from wastewater and sludge. As a result, tetracyclines may be discharged into surface waters or remain in treated sewage sludge, which can be used as a fertilizer. Additionally, the pharmaceutical industry can contribute to environmental contamination with tetracyclines. If pharmaceutical waste is not adequately treated, it may reach surface waters and soil either directly or after passing through WWTPs [59].

**Figure 4:**
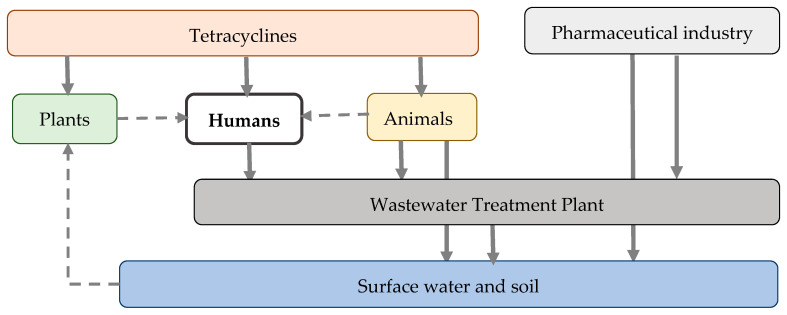
Environmental cycle of tetracyclines. Solid arrows show direct transfer of tetracyclines, whereas dashed lines represent indirect environmental exposure routes.

Tetracyclines can also be released into the environment through plant agriculture. Food crops may grow in soil containing tetracyclines or be irrigated with contaminated surface water. Moreover, plants may be exposed to tetracyclines directly for the treatment or prevention of bacterial diseases, or indirectly through fertilization with manure from livestock treated with these antibiotics or with biosolids from the WWTPs. As a result, food crops may accumulate tetracycline residues, which are subsequently ingested by humans and animals [58]. In addition to medical exposure, humans may be exposed to tetracyclines through the consumption of contaminated foods (both plant- and animal-based) and drinking water. The environmental persistence of tetracyclines promotes the development, accumulation, and dissemination of tetracycline resistance genes (*Tet genes*), contributing to AMR in human pathogens [59]. Figure 4 provides a schematic representation of the environmental cycle of tetracyclines.

### 4.2. Presence, Distribution and Concentration Levels of Tetracyclines in the Environment

There is currently no global agency that systematically monitors tetracycline residues in the environment. However, the European Commission has established the NORMAN Network (Network of reference laboratories and research centers for monitoring emerging environmental pollutants), which focuses on the surveillance, prioritization, and data sharing of contaminants of emerging concern—particularly those not yet regulated or routinely monitored. This includes antibiotics such as tetracyclines. A key component of the NORMAN network is the EMPODAT database, a geo-referenced repository that compiles occurrence data for a wide range of environmental pollutants [60]. Outside Europe, the monitoring of tetracycline residues is typically conducted at national or project-specific levels and is often fragmented and inconsistent. Tetracycline monitoring can be conducted in both aquatic matrices (e.g., surface water, groundwater, drinking water, wastewater, and biosolids) and terrestrial environments (e.g., soil and sediment).

#### 4.2.1. Tetracycline Residues in the Aquatic Environments

The EMPODAT database collects information on environmental residues of three first-generation tetracyclines: chlortetracycline, oxytetracycline, and tetracycline. For oxytetracycline, data are recorded under multiple chemical forms, including oxytetracycline, oxytetracycline dihydrate, and β-apo-oxytetracycline—a known degradation product. In the vast majority of sampling locations, tetracycline residues are reported as below the limit of quantification (LoQ) or limit of detection (LoD), indicating either trace concentrations or levels insufficient for reliable measurement [60]. Accordingly, this subsection focuses exclusively on quantifiable environmental residues, expressed in micrograms per liter (µg/L).

Since 2019, the EMPODAT database has documented quantifiable tetracycline concentrations exceeding the LoQ or LoD in three geographic localities within the European region. Although the measured concentrations ranged from 0.01 to 0.024 µg/L [60], remaining below the predicted no-effect concentrations (PNECs) for resistance selection in surface waters, they may still exert limited but non-negligible selective pressure on microbial communities [61]. A recent comprehensive review of tetracycline occurrence in aquatic environments across Europe corroborates the EMPODAT findings, noting that reported concentrations generally fall within the 0 to 0.02 µg/L range, while also providing a broader overview of country-specific data. Notably, higher concentrations were observed in wastewater-related sites, with levels reaching up to 0.238 µg/L in France [62]. Outside of wastewater-influenced settings, the highest concentrations were found in Romania, where river water samples showed levels as high as 2.45 µg/L [62], which would be classified as high relative to the PNEC thresholds [61].

In the United States, the National Water Quality Monitoring Council (NWQMC) facilitates the coordination and dissemination of environmental water quality data, including measurements of antibiotics such as tetracyclines. These data are publicly available through the Water Quality Portal (WQP), a comprehensive repository that aggregates information from multiple federal and state-level monitoring programs. Similar to EMPODAT, the WQP includes data on tetracycline-class antibiotics, such as chlortetracycline, oxytetracycline, tetracycline, and doxycycline, as well as on their environmental degradation products, including epi-, iso-, and anhydro-derivatives [63]. According to the WQP, tetracycline concentrations in most aquatic environments in the U.S. are generally at or below the LoQ or LoD. Concentrations of chlortetracycline, oxytetracycline, and doxycycline are typically low and do not exceed 0.006 µg/L. However, elevated tetracycline levels have been observed in specific locations; for example, in Pima County, concentrations reached up to 3.40 µg/L [63], exceeding PNEC thresholds and indicating potential ecological and resistance-related concerns [61].

In other countries across the Americas and the Caribbean, post-2019 data remain scarce, although studies from Brazil, Argentina, and Peru report relevant findings. The highest concentrations were observed in Argentina, where groundwater samples collected from dairy milking parlors contained up to 5.3 µg/L of tetracycline and oxytetracycline [64], a level considered high by environmental risk standards. In Brazil, untreated wastewater samples exhibited moderate concentrations ranging from 0.107 to 0.145 µg/L [65]. River water samples from Peru revealed even higher concentrations, with tetracycline levels reaching 0.201 µg/L [66].

In Asia, tetracycline concentrations in aquatic environments mostly range from 0 to 0.02 µg/L [67,68,69,70,71,72,73], although higher levels have been reported in specific locations. In India, tetracycline levels in the Ganges River reached 0.099 µg/L [67], while oxytetracycline concentrations up to 0.077 µg/L were recorded in Vietnam’s Mekong Delta [70]. Both rivers are subject to substantial anthropogenic pressures, including dense human populations and agricultural runoff. In Malaysia, a river receiving effluents from a zoo, hospital, and poultry slaughterhouse showed oxytetracycline levels of 0.109 µg/L [70]. In Bangladesh, an urban river heavily impacted by industrial and municipal discharges contained doxycycline concentrations of up to 0.19 µg/L [70]. While these concentrations remain below acute toxicity thresholds, they may exceed PNEC values relevant to resistance selection [61].

Over the past five years, the highest tetracycline concentrations globally have been reported in aquatic environments in Africa [74,75,76,77,78,79,80]. In Egypt, levels reached 3.99 µg/L in a network of drains and canals heavily impacted by industrial and domestic wastewater discharges [77]. In Nigeria, exceptionally high concentrations were recorded: up to 135.82 µg/L in river water, 23.7 µg/L in hand-dug wells, and 14.2 µg/L in bottled water [79]. These elevated concentrations are attributed to improper disposal practices, such as the dumping of expired antibiotics by drugstores and widespread trash disposal into the Osun River, which supplies local water networks. Such high levels pose significant risks by exerting strong selective pressure on environmental microbiota, thereby accelerating the spread of AMR. Table 2 summarizes tetracycline concentrations in aquatic environments globally over the past five years.

In general, as shown in Table 2, the concentrations of tetracyclines in most geographic regions are below the PNECs. However, two important considerations must be acknowledged. First, countries or regions that monitor environmental levels of tetracyclines are also more likely to have implemented bans or mitigation strategies [42]. As a result, the reported concentrations may not fully represent the situation in areas lacking surveillance, potentially underestimating the global burden. Second, emerging evidence indicates that mixtures of pharmaceuticals—even when present individually at sub-PNEC concentrations—can collectively exceed minimal selective concentrations (MSCs), thereby facilitating the selection of resistant bacteria [83]. Given that many of the studied locations are co-contaminated with other antibiotics, pharmaceuticals from diverse pharmacological classes, and heavy metals, it is plausible that both PNEC and MSC thresholds are exceeded in some environmental settings.

Terrestrial environments, such as soils, interact with aquatic ecosystems and can also accumulate tetracyclines. Since soils contain their own diverse microbial communities, the global impact of tetracycline accumulation in terrestrial environments on microbial ecology and resistance development merits consideration.

#### 4.2.2. Tetracycline Residues in the Terrestrial Environments

Compared to aquatic environments, tetracycline residues are monitored less frequently in terrestrial settings, with most research focusing on their concentrations in manure rather than in soil. However, tetracycline concentrations in manure primarily reflect their use in veterinary medicine rather than actual contamination of terrestrial environments, as not all manure is applied to land as fertilizer. For this reason, the present section focuses specifically on reported tetracycline residues in soils.

Since 2019, the EMPODAT database has included reports on tetracycline residues in soils from various European countries; however, all reported concentrations were below the LoQ or LoD [60]. Additionally, there is a lack of recent studies (from 2019 onward) reporting measured concentrations of tetracyclines in soils across Europe, with the exception of findings from the One Health European Joint Programme. In that study, tetracycline concentrations were assessed in agricultural land previously fertilized with manure, with soil samples collected from Austria, the Czech Republic, Estonia, and Portugal. Among all tetracycline-class antibiotics tested, only doxycycline was detected above the LoD, with concentrations ranging from 9.07 to 20.6 µg/kg [84]. Similarly, in the United States, the NWQMC database has reported tetracycline residues in terrestrial environments to be below the LoQ or LoD since 2019. However, individual studies have documented measurable concentrations. For example, tetracycline concentrations in soil from prairie buffer strips were found to be 7.3 µg/kg [85].

Several studies in Asia have reported tetracycline residues in soil after 2019, with the highest concentrations observed in samples collected from livestock farms in Thailand, where chlortetracycline levels reached up to 98.60 µg/kg [86]. Saudi Arabia reported the second-highest concentrations, with oxytetracycline reaching 34.52 µg/kg and doxycycline 26.54 µg/kg [87]. In other Asian countries, tetracycline residues were lower. For example, in India, chlortetracycline concentrations in agricultural soils ranged from 1.80 to 11.60 µg/kg, while oxytetracycline ranged from 0.20 to 6.50 µg/kg [88]. In China, despite soil samples also being collected from livestock farms as in Thailand, tetracycline residues were substantially lower, with the highest concentration reported for tetracycline at 1.82 µg/kg [89]. These differences may reflect variations in veterinary antibiotic usage patterns and environmental regulations among countries.

In the African region, the highest reported soil concentrations of tetracyclines were found in Egypt, where tetracycline reached 99.4 µg/kg, doxycycline 58.6 µg/kg, and oxytetracycline 56.3 µg/kg [90]. The only other African country reporting soil residues of tetracyclines was Nigeria, where tetracycline concentrations in soil near poultry farms ranged between 1.07 and 20.53 µg/kg [91]. In addition to variations in veterinary antibiotic usage and environmental regulations, differences in analytical methodologies may also account for the observed variability in tetracycline residue levels. Table 3 summarizes tetracycline residues in terrestrial environments worldwide over the past five years.

### 4.3. Harmful Effects of Tetracyclines in the Environment

Accumulation of tetracyclines in aquatic and terrestrial environments can exert direct toxic effects on non-target organisms. These effects occur even at environmentally relevant concentrations and may disrupt nutrient cycling, primary productivity, and overall ecosystem functioning [92]. In aquatic ecosystems, tetracyclines have been shown to negatively affect algae, plankton, and fish [93]. In soil environments, harmful effects have been demonstrated for soil microorganisms, earthworms, and plants, with documented impacts on microbial enzymatic activity, growth, and reproductive outcomes [94].

Tetracyclines inhibit the growth of various algae at concentrations of 0.25 mg/L and higher, with growth inhibition becoming more pronounced at higher concentrations, reaching up to 94% inhibition at 30 mg/L [95]. This inhibitory effect is attributed to the ability of tetracyclines to interfere with the protein synthesis machinery [93]. Phytoplankton and zooplankton are also susceptible to tetracycline exposure, with observed effects including reduced growth, impaired reproduction, and altered community composition [96]. In fish, tetracyclines can affect both embryos and juveniles, causing developmental abnormalities, reduced hatching success, and impaired growth [93]. Chronic exposure to oxytetracycline has been shown to induce oxidative stress, liver and kidney toxicity, and immunological changes, potentially affecting survival and reproduction [97]. Moreover, exposure to tetracyclines at concentrations of 400 mg/L has been reported to be lethal for zebrafish [98].

In terrestrial environments, tetracyclines have been shown to alter microbial community composition by reducing enzymatic activity and suppressing nitrogen and carbon cycling, thereby negatively affecting soil fertility [94]. Furthermore, these changes in microbial balance may decrease the abundance of beneficial microorganisms, such as nitrogen-fixing bacteria and mycorrhizal fungi, while potentially promoting resistant or pathogenic species [99]. Soil invertebrates, such as earthworms, also exhibit reduced growth, burrowing activity, and reproductive output, likely due to disruptions in their gut microbiome resulting from exposure to tetracyclines [94]. Plants growing in tetracycline-contaminated soils may experience impaired nutrient uptake, reduced growth, and altered root development, as a consequence of both direct antibiotic toxicity and the disruption of beneficial soil microbial communities that support plant health [99].

At sub-inhibitory concentrations, tetracyclines can exert selective pressure on microbial communities, enriching resistant bacteria and facilitating the persistence and dissemination of resistance genes. These resistant organisms and genetic elements can subsequently be transmitted to human and animal populations through environmental reservoirs, food chains, or direct contact, thereby contributing to the broader AMR crisis.

## 5. Resistance Mechanisms and Patterns

**Figure 5:**
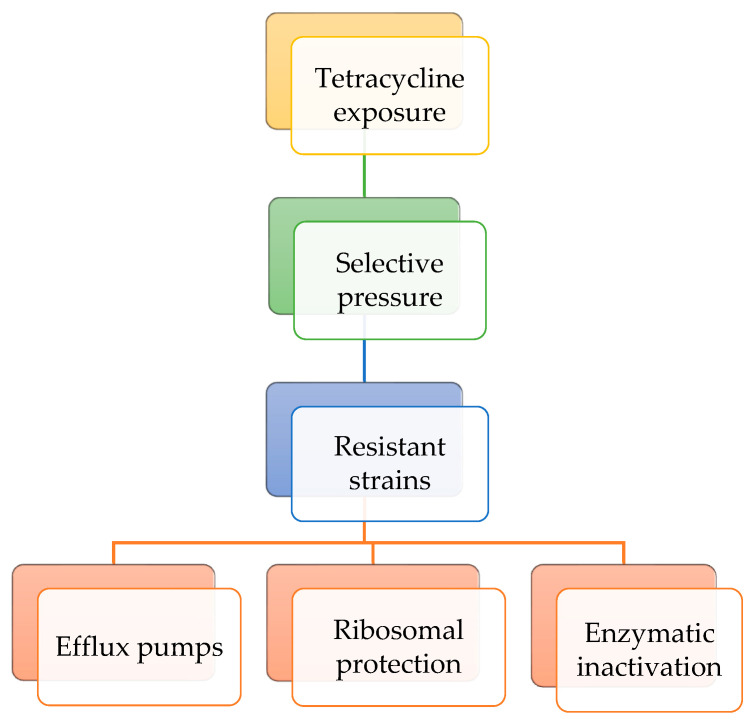
Resistance mechanisms to tetracyclines.

When bacterial populations are exposed to tetracyclines at sub-inhibitory concentrations over extended time periods, selective pressure can enrich resistant strains or induce genetic changes that enhance survival. Bacteria exhibit resistance to tetracyclines through three main mechanisms. In the efflux pump mechanism, membrane proteins actively expel tetracyclines from the bacterial cell, reducing their intracellular concentration. In the ribosomal protection level, specific proteins bind to the 30S ribosomal subunit, preventing tetracycline from binding and, in some cases, displacing the antibiotic if it is already bound. Regarding enzymatic inactivation mechanism, enzymes such as tetracycline monooxygenases chemically modify tetracyclines, rendering them inactive. These resistance mechanisms may coexist within the same bacterial strain [18]. Figure 5 presents the resistance mechanisms to tetracycline class of antibiotics.

Resistance determinants can arise through both intrinsic and extrinsic pathways. Intrinsic resistance is naturally occurring, often chromosomally encoded, and transmitted vertically from parent to daughter cells during replication. In contrast, acquired resistance arises when bacteria obtain new genetic material via horizontal gene transfer, most commonly through plasmids, but also via mobile genetic elements such as transposons and integrons [93]. A major environmental source of resistance genes is resistant gut microbiota. When resistant bacteria are excreted into the environment, they can transfer their genes to other bacteria via horizontal gene transfer. Studies have demonstrated that applying pig manure to the environment increases the abundance of antibiotic resistance genes more than cow manure, whereas the application of chicken manure leads to the highest levels of resistance genes among these livestock manures [100]. In aquatic environments, resistance genes are disseminated primarily through agricultural runoff and wastewater discharge [93].

More than 40 different tetracycline resistance genes (*tet* genes) have been identified in Gram-positive and Gram-negative bacteria. *Tet(A)*, *tet(B)*, and *tet(D)* are commonly reported in Gram-negative bacteria and encode efflux pumps, while *tet(K)* and *tet(L)* are more frequent in Gram-positive bacteria [29]. *Tet(M)*, *tet(O)*, *tet(Q)*, and *tet(W)* encode ribosomal protection proteins, whereas certain Gram-negative bacteria possess the *tet(X)* gene, which mediates enzymatic inactivation of tetracyclines [29]. Efflux pumps generally confer resistance to the older tetracyclines but are less effective against doxycycline, minocycline, and largely ineffective against third-generation agents such as tigecycline. Ribosomal protection proteins provide broader resistance, though activity against newer tetracyclines is reduced. By contrast, *tet(X)* and its variants are capable of inactivating nearly all tetracyclines, including tigecycline [29].

Humans may acquire resistant bacteria and their associated genes through direct contact with contaminated environments, consumption of food products derived from plants, animals, or fish exposed to antimicrobials, or through selective pressure from direct exposure to tetracyclines [101]. Similar to animals, the human gut microbiome can act as a reservoir, where commensal bacteria harbor and exchange resistance determinants that may subsequently transfer to pathogenic species. However, unlike animals, the contribution of the human gut microbiome to the dissemination of resistance genes into the environment is comparatively limited [101]. Nevertheless, investigation of the distribution of *tet* genes in different human populations worldwide remains as a matter of interest.

Several studies have reported the prevalence of *tet* genes and associated resistance to tetracyclines in both clinical and non-clinical settings across Europe over the past five years. Overall, the diversity of *tet* genes and the frequency of tetracycline resistance are higher in clinical settings, largely due to the more intensive and frequent exposure of patient populations to antimicrobial therapies compared with the general population. The *tet(A)* and *tet(B)* genes are among the most commonly detected in a variety of pathogens [102,103,104,105,106,107,108,109,110,111]. In some instances, resistance to tetracycline may reach 100%, as shown in a study from Italy that characterized the genetic and phenotypic features of *Streptococcus suis* isolated from cerebrospinal fluid [108]. By contrast, a study in Bulgaria examining *Enterococcus faecalis* isolated from breast milk in a non-clinical setting, that is, from healthy mothers outside of hospitals or healthcare facilities, reported a resistance rate of 22% [109]. Furthermore, a study from Spain documented resistance to tigecycline in *Klebsiella pneumoniae* and *Pseudomonas aeruginosa* strains, with prevalence rates of 10.8% and 36.3%, respectively [103].

In the Americas, the *tet(O)*, *tet(A)*, and *tet(M)* genes are the most frequently reported in isolates obtained from both clinical and non-clinical settings [112,113,114,115,116,117,118,119]. Similar to observations in Europe, isolates from patients often exhibited higher rates of tetracycline resistance, in some cases reaching 100%, such as in *Campylobacter* species isolated in Honduras [115]. Although resistance rates in isolates from healthy individuals were generally lower, they remained substantial; for instance, a study from Panama reported a 30% resistance rate with tetracycline in *Escherichia coli* [119].

*Tet(A)* and *tet(B)* genes were the most frequently reported in studies from Asia; however, several studies also documented the presence of *tet(X)* [120,121,122,123,124,125,126,127,128,129,130,131,132]. The *tet(X)* gene, a plasmid-associated determinant, confers resistance to all generations of tetracycline-class antibiotics. A study from China confirmed this by reporting 100% resistance to tigecycline, tetracycline, doxycycline, minocycline, eravacycline, and omadacycline in *Klebsiella pneumoniae* isolates from patients [120]. In contrast, a study from Kuwait identified *tet(X)* in *Enterobacteriaceae* species but found lower resistance rates to tigecycline, ranging from 0.8% to 4.1% [131]. Consistent with findings from other continents, resistance to tetracyclines in non-clinical settings was generally lower than in clinical isolates, though still considerable; for instance, a study from Lebanon reported a 42% resistance rate in *Escherichia coli* [133].

Australia and New Zealand are high-income countries with well-established AMS systems that promote the prudent use of antibiotics in both veterinary and human medicine. Reported rates of tetracycline resistance in clinical settings in Australia were 11.1% for *Campylobacter jejuni* and 2.4% for *Campylobacter coli* [134]. In New Zealand, the rates of tetracycline resistance in clinical isolates were higher, ranging from 33% to 100% in *Shigella* species [135].

*Tet(A)* and *tet(B)* genes were also the most frequently identified in isolates obtained from both clinical and non-clinical settings across countries of the African continent [136,137,138,139,140,141,142,143,144,145,146,147]. These countries report some of the highest levels of tetracycline resistance, particularly in non-clinical settings. For example, a study from Nigeria found 83% tetracycline resistance in *Escherichia coli* isolated from healthy poultry farm workers [139], while a study from Ethiopia reported resistance rates ranging from 92% to 100% in rural communities [141]. In contrast, the rates of tetracycline resistance were generally lower in urban populations, with 22.6% of urban dwellers in Ghana carrying tetracycline-resistant isolates [142], whereas higher rates were observed in disadvantaged urban communities in Kenya, where 50% of residents harbored resistant bacteria [143]. The differences in tetracycline resistance rates between rural and urban populations in Africa may be attributed to varying levels of antimicrobial use in agriculture, differences in sanitation and hygiene, and disparities in access to healthcare AMS. Table 4 provides an overview of studies reporting tetracycline resistance rates and *tet* genes isolated from humans worldwide from 2019 onward.

The global distribution of *tet* genes and the regional variation in tetracycline resistance underline the multifactorial nature of antimicrobial resistance dynamics. Several strategies can be considered to address the growing challenge of tetracycline resistance. These include the implementation of AMS programs in both human and veterinary medicine, the promotion of rational tetracycline use in agriculture, and efforts to reduce environmental contamination with these compounds. The following section provides an overview of AMS approaches relevant to the control of tetracycline resistance.

## 6. AMS Strategies to Reduce Tetracyclines’ Consumption

AMS encompasses a set of coordinated strategies and interventions aimed at promoting the appropriate use of antimicrobials in human health, veterinary medicine, and agriculture to mitigate AMR. The concept of AMS was first introduced in human medicine in the 1990s, largely in response to the growing threat of AMR within hospital settings [148]. Formal guidelines were later established in 2007 by the Infectious Diseases Society of America and the Society for Healthcare Epidemiology of America [149]. Following its adoption in human medicine, the principles of AMS were extended to veterinary practice in the early 2000s. The WOAH issued its first recommendations in 2003, integrating AMS principles into international standards for the prudent use of antimicrobials in animals [44]. In agriculture and aquaculture, AMS gained increasing recognition in the 2010s, particularly after the FAO released its Action Plan on AMR in 2016 [150]. In 2015, the WHO launched the Global Action Plan on AMR, which unified AMS efforts under the One Health framework, emphasizing the interconnectedness of human, animal, and environmental health [151].

### 6.1. AMS Strategies to Reduce Tetracyclines Consumption in Human Medicine

In human medicine, AMS strategies are guided by five overarching goals. The first is to raise awareness among healthcare providers and promote adherence to the “5Ds of antimicrobial therapy”: prescribing the right Drug, at the correct Dose, via the appropriate Delivery route, for the optimal Duration, and with suitable De-escalation of therapy when possible [148]. The second one is to prevent the misuse and overuse of antimicrobials in both hospital and community healthcare settings, as well as in veterinary medicine, agriculture, and aquaculture. The third, fourth, and fifth goals are to reduce the incidence of antibiotic-associated infections such as *Clostridioides difficile*, minimize AMR, and decrease healthcare-related costs, respectively [148].

To guide AMS, the WHO introduced the AWaRe classification in 2017 as part of the Model List of Essential Medicines. This framework groups antibiotics into three categories—“Access”, “Watch”, and “Reserve”—based on their spectrum of activity, potential for resistance development, and clinical importance in human medicine. Antibiotics in the “Access” group have a lower potential for resistance selection and are recommended as first- or second-line treatments for common infections. The “Watch” group includes antibiotics with a higher risk of resistance and should be prioritized for specific, limited indications. The “Reserve” group comprises last-resort antibiotics, intended for use in treating confirmed or suspected infections caused by multidrug-resistant pathogens [152].

In 2019, the WHO updated the AWaRe classification, substantially expanding the list of antibiotics included and providing clearer guidance for monitoring antibiotic use at national and global levels [153]. The updated framework also established specific global targets to promote the rational use of antimicrobials. In the same year, the WHO set a goal for antibiotics in the “Access” group to account for at least 60% of total antibiotic consumption, implying that the combined use of “Watch” and “Reserve” group antibiotics should not exceed 40% [154]. In 2024, this target was revised, with the WHO aiming for “Access” group antibiotics to constitute at least 70% of total antibiotic consumption by 2030 [155].

The WHO also developed the Model List of Essential Medicines (EML) and the Model List of Essential Medicines for Children (EMLc), which serve as global references for the selection of safe, effective, and cost-efficient medicines that address the most pressing public health priorities, including AMR [156]. Together, the AWaRe classification and the EML/EMLc lists provide a comprehensive framework for promoting AMS strategies.

According to the latest edition of the WHO AWaRe classification, only two tetracyclines (tetracycline and doxycycline) are categorized as “Access” group antibiotics. Chlortetracycline, oxytetracycline, demeclocycline, penimepicycline, clomocycline, lymecycline, rolitetracycline, metacycline, and sarecycline are classified under the “Watch” group, while tigecycline, omadacycline, and eravacycline are categorized as “Reserve” group antibiotics. Oral minocycline is assigned to the “Watch” group, whereas intravenous minocycline is classified as a “Reserve” antibiotic [157]. At the same time, according to the latest EML, doxycycline is the only tetracycline-class antibiotic listed for systemic use, while chlortetracycline, oxytetracycline, and tetracycline are included for topical use in ophthalmic preparations [156]. Table 5 provides an overview of these categorizations.

Beyond the global-level lists, many countries develop their own national antibiotic lists tailored to local epidemiological patterns, healthcare priorities, and resource availability. These national lists may differ from the WHO classifications and categorize tetracycline antibiotics differently. For instance, countries with a high incidence of zoonotic transmission of *Brucella* spp. may designate tetracycline as a critically important antibiotic, given the limited availability of effective alternatives against endemic brucellosis [158]. This underlines the importance of an integrated One Health approach for implementing AMS strategies across human and veterinary medicine.

### 6.2. AMS Strategies to Reduce Tetracyclines Consumption in Veterinary Medicine, Agriculture, and Food Systems

Similar to the WHO, the WOAH has established a set of principles to promote the prudent, safe, and effective use of antimicrobials in veterinary medicine. These principles are detailed in the Terrestrial Animal Health Code [159] and the Aquatic Animal Health Code [160]. A central principle emphasized in these documents is the prohibition of antimicrobial use as growth promoters unless a comprehensive risk analysis demonstrates that such use does not pose a threat to human health. Nevertheless, according to the most recent WOAH Annual Report on Antimicrobial Agents Intended for Use in Animals, 22% (34 out of 157) of member countries still reported using antimicrobials for growth promotion purposes [48]. In addition, both Codes stipulate prescription-only access to veterinary antimicrobials, mandate surveillance and data reporting on antimicrobial use in animals, and emphasize the promotion of alternatives to antimicrobials, such as probiotics and vaccines [159,160].

The WOAH has also aligned its efforts with the WHO by developing a List of Antimicrobial Agents of Veterinary Importance, which classifies antimicrobials into three categories: “veterinary critically important antimicrobial agents” (VCIA), “veterinary highly important antimicrobial agents” (VHIA), and “veterinary important antimicrobial agents” (VIA). This list includes four tetracyclines (chlortetracycline, doxycycline, oxytetracycline, and tetracycline) all categorized as VCIA. The rationale for this classification is that tetracyclines have extensive applications in veterinary medicine, treating both bacterial and chlamydial infections across a wide range of animal species. Moreover, they remain the only effective antibacterials for the treatment of heartwater, caused by *Ehrlichia ruminantium*, and anaplasmosis, caused by *Anaplasma marginale* [161]. Table 6 summarizes the key provisions regarding the use of tetracyclines in veterinary medicine as reflected in the WOAH list of antimicrobial agents of veterinary importance.

The FAO has also developed a series of guidelines and best-practice codes aimed at reducing and optimizing antimicrobial use in agriculture and throughout the food chain. For example, in collaboration with the WHO, the FAO formulated the Code of Practice to Minimize and Contain Foodborne AMR, which promotes the responsible and prudent use of antimicrobials. This document encourages the gradual phase-out of non-therapeutic applications and advocates for enhanced hygiene, biosecurity, and vaccination measures to reduce the need for antibiotic use [162]. Furthermore, the FAO’s Action Plan on AMR focuses on strengthening governance and regulatory capacity in food and agriculture, advancing good agricultural and veterinary practices, and fostering research into alternatives to antibiotics [163]. Although the FAO supports the progressive reduction of antibacterial use in the agricultural sector, some high-income countries, including the United States, members of the EU, and Japan, continue to regulate the use of plant-related antimicrobial pesticides, among which tetracyclines remain permitted as bactericides [53].

### 6.3. One Health Approach

Although the conceptual origins of the One Health approach can be traced back to the 19th century, when the interconnections between human and animal diseases were first recognized, the modern One Health movement emerged in the early 2000s, following outbreaks of major zoonotic diseases such as avian influenza (H5N1), severe acute respiratory syndrome, and Middle East respiratory syndrome [164]. In response to these challenges, the WHO, the WOAH, and the FAO established the Tripartite Collaboration, which expanded into the Quadripartite Alliance in 2022 with the inclusion of the United Nations Environment Programme (UNEP). The Quadripartite One Health Joint Plan of Action (2022–2026) outlines strategic objectives aimed at strengthening global surveillance, improving policy coherence, and enhancing capacity building across sectors [165].

In alignment with these efforts, the WHO developed the List of Medically Important Antimicrobials (MIA) to categorize antimicrobial agents used in both human and veterinary medicine based on their relevance to human health. According to this classification, antimicrobials are divided into two main groups: “medically important” (those used in human medicine) and “not medically important” (those not authorized for human use). The “medically important” antimicrobials are further divided into those authorized exclusively for human medicine and those permitted for use in both human and veterinary medicine. Among the tetracycline class, fluorocyclines (eravacycline), aminomethylcyclines (omadacycline), and glycylcyclines (tigecycline) are restricted to human use. However, the MIA classification recommends excluding these compounds from the tetracycline class due to distinct resistance mechanisms [166].

Antimicrobials authorized for both human and veterinary use are subdivided into four categories: “critically important antimicrobials”, “highly important antimicrobials”, “highest priority critically important antimicrobials”, and other MIA. Within this framework, the majority of tetracycline antibiotics are classified as “highly important antimicrobials” because there are few alternative agents with proven efficacy against *Brucella* spp., *Chlamydia* spp., and *Rickettsia* spp. [166]. Table 7 summarizes the classification of tetracycline-class antibiotics according to the MIA framework.

## 7. Degradation Strategies

AMS strategies help reduce tetracycline consumption; however, they do not address the persistence of tetracyclines already present in the environment. These compounds tend to accumulate in soil and aquatic systems, where they continue to exert selective pressure on microbial communities, thereby promoting AMR. Although tetracyclines are relatively stable under environmental conditions, various physicochemical degradation techniques have been developed to exploit their specific structural and chemical characteristics [18].

Tetracycline degradation occurs through several distinct mechanisms, each involving characteristic structural transformations of the tetracycline scaffold [79]. Hydrolytic degradation primarily affects the amide and lactone functional groups, leading to cleavage of the C–N and C–O bonds and formation of less active ring-opened products [92]. Photodegradation is initiated by light-induced excitation of the conjugated aromatic system, promoting tautomerization, hydroxylation, demethylation, and oxidative cleavage of the dimethylamino group at C4, ultimately generating a series of epimers, anhydro-derivatives, and smaller aromatic fragments [79]. Oxidative degradation, including advanced oxidation processes, typically proceeds through hydroxyl radicals and other reactive oxygen species (ROS) that attack the phenolic D-ring, causing hydroxylation, dealkylation, and fragmentation of the polycyclic structure [93]. Biodegradation involves microbial enzymatic pathways—such as deamination, decarboxylation, and ring-cleavage reactions—producing simpler organic acids, alcohols, and CO_2_ [92]. Together, these mechanisms reduce tetracycline bioactivity and influence their persistence, mobility, and ecotoxicity in the environment.

Tetracycline metabolites, produced through environmental degradation, can exhibit toxicity and affect aquatic organisms. For example, they have been shown to impair the growth and photosynthesis of freshwater algae [167]. Although sometimes less active than the parent compound, these metabolites persist in water and soil, posing ecological risks by impacting microbial communities and higher trophic levels [168]. Thus, the environmental effects of tetracyclines should consider both the parent drugs and their potentially toxic metabolites.

### 7.1. Degradation of Tetracyclines via Hydrolysis, Photodegradation, and Oxidative Degradation

Degradation of tetracyclines through hydrolysis is significantly influenced by environmental factors such as pH and temperature. Extreme pH values and elevated temperatures accelerate the process. For instance, the hydrolysis rate has been reported to increase by 2.22- to 2.74-fold with every 10 °C rise in temperature [169]. Conversely, lower ambient temperatures markedly reduce the rate of hydrolysis; at 4 °C, more than 90% of tetracycline remained unchanged after 48 h [170]. In addition to temperature, pH strongly affects the hydrolysis rate, with alkaline conditions promoting hydrolysis more efficiently. For example, the rate of oxytetracycline hydrolysis was 72.7% at pH 6.91, but only 10.5% at pH 3.09 [171]. Furthermore, the presence of certain metal ions, such as calcium and magnesium, can influence hydrolysis kinetics, as tetracyclines are known to form chelate complexes with these cations [172]. The primary hydrolysis products are epimers and anhydro-tetracyclines [169]. Several of these transformation products retain significant biological activity and, in some cases, exhibit greater toxicity than the parent compound. For example, 4-epitetracycline and anhydro-tetracycline have been shown to demonstrate enhanced cytotoxic effects and may contribute to gastrointestinal irritation and nephrotoxicity. Thus, although hydrolysis can reduce the concentration of the parent compound, it does not necessarily eliminate ecological or toxicological risks due to the persistence and biological activity of the resulting degradation products [173].

Similar to hydrolysis, the photodegradation of tetracyclines is affected by pH, temperature, light intensity, wavelength, and the presence of photosensitizers. In general, alkaline conditions enhance photodegradation due to increased formation of ROS and photoactive anionic forms of tetracyclines, while acidic conditions tend to slow the process because of reduced light absorption and the predominance of less reactive protonated species [76]. For example, the photocatalytic oxidation efficiency of tetracycline increased from 75.8% to 86.3% when pH rose from 4 to 10 [174]. The optimal pH range for photocatalytic degradation is typically 8–9, whereas strongly alkaline conditions (pH 11) may inhibit the process [175]. Temperature also affects photodegradation: higher temperatures enhance molecular mobility and reaction kinetics, leading to faster degradation, although its effect is secondary to that of light intensity, wavelength, or pH. For example, studies have shown that tetracycline photodegradation proceeds more rapidly at 60–80 °C than at 45 °C [176]. The degradation rate also depends on the wavelength and intensity of light. Shorter wavelengths promote faster photodegradation due to higher photon energy, while greater irradiance increases the number of photoexcited electrons and holes, thereby generating more ROS and accelerating degradation [177]. Moreover, environmental photosensitizers such as humic substances and nitrogen oxides can further enhance photodegradation by facilitating ROS production [178]. The photodegradation of tetracyclines can lead to the formation of numerous transformation products, including epimers, iso-tetracyclines, anhydro-derivatives, and various hydroxylated and ring-cleaved intermediates. Several of these photoproducts exhibit equal or greater toxicity compared with the parent compound. For example, anhydro- and iso-tetracycline derivatives have been associated with increased phototoxicity, enhanced cytotoxic effects, and potential hepatotoxicity. Moreover, some photoproducts also retain antimicrobial activity, contributing to sustained selective pressure in aquatic environments [92].

Ozone, hypochlorous acid, hydrogen peroxide, peroxymonosulfate, and persulfate are commonly used oxidants for the oxidative degradation of tetracyclines. Similar to hydrolysis and photodegradation, the efficiency of oxidative degradation depends on environmental factors such as pH, initial tetracycline concentration, and the presence of interfering substances. Lower pH generally enhances oxidation, with the highest degradation rate constant observed at pH 4. As pH increases, the rate decreases, reaching its minimum at pH 12, which is approximately 1/38 of that observed at pH 4 [179]. Regarding the influence of initial tetracycline concentration, the maximum degradation rate constant was reported at 5 mg/L, while further increases in concentration led to progressively slower degradation, with the rate constant at 100 mg/L reduced to about 1/76 of that at 5 mg/L [179]. The presence of natural organic matter such as humic acids and other dissolved organic compounds can either enhance or inhibit oxidative degradation. For example, in a peroxymonosulfate/LaCoO_3_ system, tetracycline degradation reached 90% within 30 min in the absence of coexisting substances, while low concentrations of humic acid slightly accelerated the process [180]. Conversely, other studies have shown that humic acid can inhibit tetracycline degradation by scavenging reactive radicals and reducing intermediate oxidation products back to their original state [181]. Several oxidative transformation products of tetracyclines (including anhydro- and epimeric derivatives) have been shown to retain antimicrobial activity and may induce oxidative stress in algae and other biota [92]. For example, UV/PS-generated by-products inhibited algal growth and triggered antioxidant enzyme responses in *Chlorella* [182].

In current environmental and engineering contexts, photodegradation primarily governs the fate of tetracyclines in surface environments, whereas oxidative processes are employed in wastewater treatment systems for rapid and near-complete removal. At the same time, hydrolysis remains a slower, natural attenuation pathway that contributes mainly to partial degradation [92]. In recent years, biodegradation approaches, using bacteria, fungi, and enzyme-based systems, have been proposed as environmentally sustainable alternatives and represent an active area of ongoing research.

### 7.2. Biodegradation of Tetracyclines

Biodegradation of tetracyclines is an area of active investigation, particularly as a sustainable alternative to physicochemical degradation techniques. It can proceed through two main pathways: (1) biotransformation, in which the antibiotic molecule is structurally modified or detoxified through enzymatic reactions, and (2) mineralization, involving the complete breakdown of the compound into inorganic end products such as carbon dioxide, water, and mineral salts [183]. Biodegradation may be carried out by a single microbial strain, commonly belonging to the *Proteobacteria*, *Actinobacteria*, or *Firmicutes* phyla, or by microbial consortia that coexist in environments such as soil, activated sludge, or livestock manure [184]. The use of mixed microbial communities often enhances the degradation efficiency because of the synergistic interactions between species capable of attacking different structural moieties of tetracycline molecules. For example, studies have shown that co-cultures outperform monocultures in tetracycline/oxytetracycline biodegradation, likely due to complementary enzymatic activities [185]. Fungi, like yeasts, are also capable for tetracycline degradation [186].

Similar to physicochemical degradation techniques, the biodegradation of tetracyclines is also influenced by environmental factors such as temperature, pH, and the presence of other substances. For instance, the degradation half-life of oxytetracycline decreases with increasing pH (from 5.5 to 7.4) [187]. Likewise, oxytetracycline degradation is more pronounced at higher temperatures (23.0 °C and 31.2 °C) compared with lower temperatures (1.8 °C) [187]. Elevated temperatures, such as those occurring during composting, can further enhance the degradation of residual tetracyclines. For example, the degradation rate of tetracyclines in swine manure increased with rising temperature and reached its maximum at 55 °C, likely due to a combined effect of biodegradation and thermal degradation [188]. When tetracyclines are degraded by yeasts, temperature also plays a critical role, with the highest biodegradation efficiency (85.1%) observed at 35 °C, followed by 40 °C and 30 °C [186].

The presence of other substances, such as easily biodegradable organic matter or catalysts, can enhance tetracycline biodegradation or affect the overall removal efficiency. Co-existing organic matter, including simple carbon sources like glucose or acetate, may stimulate tetracycline removal by promoting the growth and metabolic activity of degrading microorganisms. Additionally, humic substances and natural organic matter can facilitate the sorption of tetracyclines, thereby modifying their bioavailability and degradation kinetics [189]. In soil environments, earthworms have been shown to accelerate tetracycline degradation by increasing soil pH and dissolved organic carbon concentrations [190]. Catalytic materials, such as biochar, metal oxides, and clay minerals, can also influence biodegradation by enhancing adsorption processes that concentrate the antibiotic and create nutrient-rich microenvironments favorable for microbial activity [191]. However, excessive levels of co-existing organic compounds or metals may inhibit enzymatic activity or induce microbial competition, ultimately reducing the overall degradation efficiency of tetracyclines [192]. Studies have reported that certain biodegradation intermediates can inhibit algal growth, induce oxidative stress in aquatic microorganisms, and alter microbial community composition due to their residual bioactivity [193]. Consequently, biologically active metabolites of tetracycline biodegradation may persist in the environment, maintaining ecological and toxicological risks.

**Figure 6:**
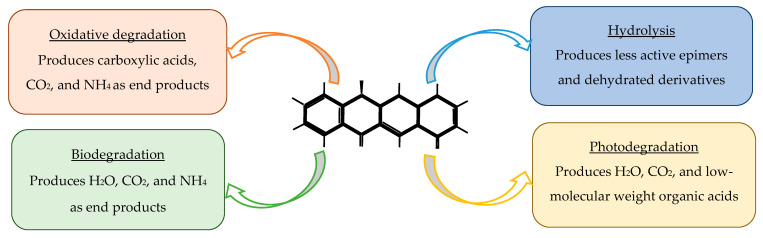
Environmental degradation pathways of tetracyclines.

Nevertheless, it should be noted that despite substantial progress in developing various tetracycline degradation techniques, most approaches remain confined to laboratory-scale studies and have limited translation into real-world environmental or engineering applications. Hydrolysis, photodegradation, and biological degradation occur naturally and contribute significantly to tetracycline degradation under environmental conditions [194]. In contrast, oxidative degradation methods face practical challenges, including high operational costs, complex process optimization, and limited scalability [195]. These limitations highlight the need for further research aimed at optimizing such technologies for field implementation and assessing their long-term environmental performance. Figure 6 provides an overview of the currently available tetracycline degradation techniques.

### 7.3. Additional Removal and Treatment Technologies for Tetracyclines

In addition to chemical and biological degradation processes, various treatment technologies have been developed to remove tetracyclines from aquatic and terrestrial environments. Adsorption is one of the most widely applied approaches, using materials such as activated carbon, biochar, clays, metal–organic frameworks, and graphene-based materials, which can efficiently bind tetracyclines through electrostatic interactions, π–π stacking, and surface complexation. These materials offer an efficient, often low-cost, and environmentally friendly method for treating wastewater contaminated with antibiotics [196].

Membrane filtration techniques like nanofiltration and reverse osmosis are highly effective at removing contaminants but face challenges with membrane fouling and high energy consumption. While reverse osmosis can provide very high removal efficiencies, nanofiltration may be a more energy-efficient alternative for certain applications [197]. Constructed wetlands and biofiltration systems are increasingly used to sustainably remove tetracycline from water by using natural processes like plant–microbe interactions and substrate adsorption. These systems act as a low-energy option that uses a combination of biological breakdown by microbes, physical removal through filtration, and chemical processes like adsorption and precipitation to eliminate tetracyclines [198]. These removal technologies complement degradation pathways and form part of an integrated strategy for mitigating tetracycline contamination in the environment. Table 8 summarizes the advantages and disadvantages of different degradation methods.

In addition to efficiency and environmental considerations, economic and energy requirements are important factors when evaluating tetracycline degradation methods. Chemical oxidation tends to be energy-intensive and incurs higher operational costs due to the need for reagents, catalysts, and specialized equipment [199]. Photodegradation, particularly when using high-intensity light sources, also increases energy demand and associated operational costs [200]. Hydrolysis is relatively low-cost and passive, making it energy-efficient, but it is limited by long reaction times and strong dependence on environmental conditions [173]. Adsorption processes are generally economical and energy-efficient; however, the need for regeneration or replacement of adsorbents contributes to long-term costs [196]. Similarly, biodegradation is typically low-energy and cost-effective, but degradation rates are slow and may require large treatment areas [183]. Overall, among these methods, biodegradation and adsorption are more feasible for large-scale, low-cost applications, whereas oxidative and photochemical treatments are better suited for the rapid degradation of highly concentrated effluents, albeit at higher economic and energy costs [93].

## 8. Future Directions

The findings summarized in this review indicate two major directions for future work: research and policy development. Tetracyclines remain essential for human and veterinary medicine, as well as for agricultural and aquacultural applications; therefore, their preservation is critical in addressing AMR within this antibiotic class. In addition to the ongoing search for novel tetracycline derivatives, a recommendation applicable to other antibiotic classes as well [201], the prudent use of existing tetracycline agents must be ensured through the implementation of coordinated and evidence-based strategies.

In the context of human medicine, many countries and territories remain underinvestigated with respect to tetracycline consumption during the period from 2019 to the present. Although the ECDC [33] and the WHO Regional Offices for Europe [34] and the Western Pacific [35] collect antibiotic consumption data and publish annual reports for their member states, such systematic monitoring does not extend to other parts of the world. The African continent remains the largest blind spot on the global map of tetracycline consumption, despite Tanzania reporting the highest global rate (17.0 DDD in 2019) [39]. The reasons for this exceptionally high figure require further investigation, and the reported consumption in Tanzania’s human healthcare sector should be independently validated. A possible explanation may relate to the high prevalence of zoonotic infections such as *Brucella* spp., for which tetracyclines are classified as critically important antibiotics [202]. Establishing robust and reliable national surveillance systems for antibiotic consumption is essential to support the effective implementation of AMS strategies [203].

Even less is known about tetracycline consumption in the animal health sector, despite this antibiotic class being the most widely used in veterinary medicine. Although 85 countries are members of the WOAH, an even smaller number report data on tetracycline use to the ANIMUSE platform [44]. While available evidence suggests that Canada has among the highest reported tetracycline consumption rates, this finding should be interpreted with caution due to the limited availability of data from most countries in Asia, Africa, and Latin America. Given that the use of tetracyclines for growth promotion is now banned in many regions, further research is needed to quantify their therapeutic use and to assess national policies promoting their rational use in the animal health sector [204]. In agriculture and aquaculture, no international organization systematically collects data on tetracycline consumption, leaving the true scale of the problem largely unknown. Therefore, establishing reliable consumption estimates represents a critical first step toward developing evidence-based policy interventions in these sectors [205]. This aligns with the One Health framework and contribute directly to achieving global targets under the WHO AMR Action Plan and the UN Sustainable Development Goals on health and clean water.

Significant knowledge gaps remain regarding the environmental occurrence of tetracyclines over the past five years, as no international agency systematically collects such data. The information available in the EMPODAT database [60] is largely outdated, with most records on tetracycline pollution referring to earlier time periods. Another limitation is the predominant focus of existing studies on tetracycline concentrations and removal efficiency in WWTPs. However, it is equally important to assess tetracycline levels in aquatic environments, particularly in water bodies used as sources for human and animal consumption [206]. Furthermore, data on tetracycline contamination in terrestrial ecosystems are scarce, with most reports limited to manure and soils surrounding livestock farms. Understanding the extent of tetracycline pollution in other terrestrial environments, especially those used for animal feeding and crop production, is essential for comprehensive environmental risk assessment [207].

Although numerous studies have reported the prevalence of tetracycline resistance and the distribution of various *tet* genes in humans, most of these investigations are based on clinical samples collected from patients with diverse infections. This makes it challenging to determine the extent to which observed resistance patterns are attributable to prior environmental exposure to tetracyclines or to recent antibiotic therapy [18]. Therefore, additional studies assessing the prevalence of resistance to different tetracycline-class antibiotics, as well as *tet* genes, in healthy populations are needed. Of particular importance is the surveillance of resistance to tigecycline, minocycline, omadacycline, and eravacycline, four tetracyclines classified within the “Reserve” group [153], along with the monitoring of *tet(X)* genes involved in horizontal gene transfer that can confer resistance to these agents [208]. Such surveillance should be conducted at the country or subregional level, as resistance patterns and the dissemination of *tet* genes tend to vary geographically due to differences in antibiotic usage practices and local microbial ecology [209].

Among the currently available degradation techniques, oxidative processes, particularly advanced oxidation processes, show considerable promise, as they are capable of degrading tetracyclines into smaller and less toxic molecules within relatively short timeframes [210]. Despite their high efficiency, large-scale implementation of oxidative degradation technologies remains limited due to high operational costs, the need for precise chemical dosing, and the complexity of maintaining optimal reaction conditions in real-world environmental settings [211]. Future translational research should aim to optimize these methods for broader environmental applications by incorporating low-cost catalysts, renewable energy sources, and hybrid treatment systems that integrate oxidation with biological or adsorption-based processes [212]. In particular, a focus needs to be made on developing green, energy-efficient degradation methods, such as solar-driven photocatalysis and biochar-assisted hybrid systems, to reduce environmental impact and operational costs [213]. At the agricultural level, particular emphasis should be placed on developing scalable manure treatment and management strategies, including advanced approaches such as composting with optimized microbial consortia, anaerobic digestion, and integrated thermal or oxidative treatment technologies [214].

Moving forward, a multidisciplinary approach integrating microbiology, environmental science, and health policy is essential to address the complex challenges posed by tetracyclines. Linking antibiotic consumption data with environmental monitoring and resistance surveillance will enable the development of predictive models to assess AMR risks. In parallel, the translation of laboratory-based degradation technologies into field-scale applications requires joint efforts from chemists, engineers, and policymakers to ensure economic feasibility and environmental sustainability. Establishing international platforms for data sharing and harmonized surveillance will be key to guiding evidence-based interventions and promoting the responsible use of tetracyclines across sectors.

## 9. Conclusions

Tetracyclines constitute an important class of antibiotics with broad applications in human and animal health, as well as in agriculture and aquaculture. Due to their extensive use in veterinary medicine, the emergence and dissemination of AMR mediated by *tet* genes pose a significant threat to human health, potentially rendering last-resort antibiotics from this class ineffective. Substantial knowledge gaps persist regarding tetracycline consumption across sectors and the contamination of aquatic and terrestrial environments with these compounds—information that is essential for the development of effective AMS and environmental remediation strategies. Although numerous degradation techniques have been proposed, their application remains largely confined to engineered or laboratory conditions, underscoring the need for translational research to facilitate implementation in real-world settings. Coordinated efforts by international and national regulatory bodies are required to design and enforce targeted actions informed by local contexts, thereby ensuring the long-term preservation of the therapeutic effectiveness of this important antibiotic class.

## Figures and Tables

**Table 1:** Spectrum of antibacterial activity of tetracyclines against major bacterial genera.

Category	Representative Genera/Species
Gram-positive bacteria	*Actinomyces*, *Bacillus*, *Listeria*, *Streptococcus*, *Staphylococcus*, *Cutibacterium acnes*, *Clostridium* spp., *Rothia* spp., *Helcococcus* spp., *Nocardia*.
Gram-negative bacteria	*Escherichia coli* (enterotoxigenic), *Haemophilus*, *Moraxella*, *Neisseria*, *Pasteurella*, *Vibrio*, *Yersinia*, *Aeromonas* spp., *Burkholderia*, *Acinetobacter* spp. (including *A. baumannii* with some sensitivity to tigecycline), *Stenotrophomonas maltophilia* (sensitive to minocycline).
Atypical and intracellular bacteris	*Chlamydia*, *Chlamydophila*, *Mycoplasma*, *Ureaplasma*, *Rickettsia*, *Ehrlichia*, *Anaplasma*, *Coxiella*, *Orientia*, *Wolbachia*
Spirochetes and related genera	*Borrelia*, *Treponema*, *Leptospira*, *Brachyspira*, *Spiroplasma*
Zoonotic and other pathogens	*Brucella*, *Bartonella*, *Francisella*, *Legionella*, *Campylobacter*, *Helicobacter*, *Tropheryma whipplei*

**Table 2:** Prevalence of tetracycline residues in aquatic environments worldwide.

World Region	Molecule	Country	Type of Water Matrix	Quantity, µg/L	Reference
Europe	Chlortetracycline	The Netherlands	River	0.02	[60]
	Tetracycline			0.015	
		Czech Republic	* WWTP	0.096	[62]
		France	WWTP	0.238	
			Surface water	0.001–0.055	
			Groundwater	0.01–0.049	
		Poland	River	0–0.0074	
	Oxytetracycline	Ukraine		0.024	[60]
		Poland		0.0007–0.175	[62]
		Romania		0.034–2.45	
		Greece	Seawater	0.0126–0.0844	[81]
	Doxycycline	France	^ UTW	0.007	[62]
		Poland	River	0–0.132	
Americas	Chlortetracycline	United States		0.006	[63]
	Tetracycline			0–3.40	
		Brazil	WWTP	0.107–0.145	[65]
		Argentina	Groundwater	0.1–5.3	[64]
		Peru	River	0.201	[66]
	Oxytetracycline	Argentina	Groundwater	0.1–5.3	[64]
		United States	River	0.002–0.006	[63]
	Doxycycline				
Asia	Chlortetracycline	China	River	0.0232	[67]
		Myanmar/Thailand coastline	Seawater	0.00004–0.003	[71]
	Tetracycline	India	River	0.00125–0.099	[82]
		China	WWTP	0.0036–0.036	[68]
		Iraq	River	0.002–0.0436	[69]
		Indonesia	Reservoir	0–0.005	[73]
		Myanmar/Thailand coastline	Seawater	0.00006–0.005	[71]
	Oxytetracycline			0.00012–0.013	
		Pakistan	Groundwater	0.0418	[72]
		China	WWTP	0.0068–0.088	[68]
		Malaysia	River	0.02–0.109	[70]
		Vietnam		0.023–0.077	
	Doxycycline				
		China	WWTP	0.0074–0.0251	[68]
		Bangladesh	River	0–0.19	[70]
	Tigecycline	Pakistan	Groundwater	0.02832	[72]
Africa	Chlortetracycline	Malawi	River	0.0028–0.0055	[76]
		Uganda	Groundwater	0.0078–0.0922	[80]
	Tetracycline	South Africa	WWTP	0.0001–0.00024	[74]
		Kenya	River	0.3	[75]
		Egypt	Drain/Canal	0.28–3.99	[77]
		Nigeria	River	3.41–135.82	[79]
			Well water	7.22–23.7	
			Bottle water	0–14.2	
		Uganda	Groundwater	<0.0064	[80]
	Oxytetracycline			<0.0015	
	Doxycycline	South Africa	WWTP	0.00538–0.12	[74]
		Malawi	River	0–0.0021	[76]
		Ghana	Hospital wastewater	0.00133–0.00293	[78]

* WWTP—wastewater treatment plant. ^ UTW—urban treated wastewater.

**Table 3:** Prevalence of tetracycline residues in terrestrial environments worldwide.

World Region	Molecule	Country	Type of Soil	Quantity, µg/kg	Reference
Europe	Doxycycline	Austria, Czech Republic, Estonia, Portugal	Agricultural land	9.07–20.6	[84]
Americas	Tetracycline	United States	Prairie soil	7.3	[85]
Asia	Chlortetracycline	China	Livestock farm	0.125	[89]
		India	Agricultural land	1.80–11.60	[88]
		Thailand	Livestock farm	35.5–98.60	[86]
		Saudi Arabia	Dryland soil	9.35	[87]
	Tetracycline			14.94	
		Thailand	Livestock farm	1.87–9.33	[86]
		China		1.82	[89]
	Oxytetracycline			0.17	
		Thailand		2.67–10.80	[86]
		India	Agricultural land	0.20–6.50	[88]
		Saudi Arabia	Dryland soil	34.52	[87]
	Doxycycline			26.54	
		China	Livestock farm	0.755	[89]
		Thailand		12.22	[86]
		India	Agricultural land	0.30–0.80	[88]
Africa	Chlortetracycline	Egypt		18.1–79.0	[90]
	Tetracycline			14.2–99.4	
		Nigeria	Soil around poultry farm	1.07–20.53	[91]
	Oxytetracycline	Egypt	Agricultural land	20.5–56.3	[90]
	Doxycycline			18.5–58.6	

**Table 4:** Prevalence of tetracycline resistance and distribution of *tet* genes in bacterial isolates obtained from human clinical and non-clinical samples worldwide.

World Region	Country	Sample Origin	Pathogen	Resistance Genes	Tetracycline Resistance Rates (%)	Reference
Europe	Austria	Clinical samples from patients	*Escherichia coli*	Not specified	Tetracycline (18.3)	[102]
			*Enterococcus faecalis*		Tetracycline (33.3)	
	Spain		*Acinetobacter baumannii*	*Tet(A)*, *tet(B)*, *tet (E)*	Tigecycline (* N/D)	[103]
			*Escherichia coli*	*Tet(A)*, *tet(B)*		
			*Klebsiella pneumoniae*	*Tet(A)*, *tet(B)*, *tet (D)*, *tet (E)*	Tigecycline (10.8)	
			*Pseudomonas aeruginosa*	*Tet(A)*, *tet(B)*, *tet (E)*	Tigecycline (26.3)	
	Hungary		*Acinetobacter baumannii*	*Tet(B)*	** N/R	[104]
	Croatia		*Proteus mirabilis*	*Tet(A)*, *tet(C)*, *tet(H)*, *tet(J)*		[105]
	Armenia		*Klebsiella pneumoniae*	*Tet(A)*		[106]
	Slovenia	Blood	*Methicillin-resistant Staphylococcus aureus*	*Tet(K)*, *tet(M)*	Tetracycline (8.5)	[107]
	Italy	Cerebrospinal fluid	*Streptococcus suis*	*Tet(W)*	Tetracycline (100.0)	[108]
	Bulgaria	Breast milk	*Escherichia faecalis*	*Tet(M)*, *tet(S)*	Tetracycline (22.0)	[109]
	Romania	Feces	*Campylobacter jejuni*	*Tet(O)*	Tetracycline (55.0)	[110]
	Greece		*Salmonella enterica*	*Tet (A)*	Tetracycline (N/R)	[111]
Americas	Chile	Clinical samples from patients				[112]
	Brazil		*Streptococcus agalactiae*	*Tet(M)*, *tet(O)*, *tet (S)*	Tetracycline (80.2)	[113]
	USA		*Neisseria gonorrhoeae*	*Tet(M* *)*	Tetracycline (N/R), doxycycline (N/R)	[114]
	Honduras		*Clostridium difficile*		Tetracycline (100.0)	[115]
	Peru	Feces	*Campylobacter species*	*Tet(O)*	Tetracycline (76.0)	[116]
	French Guiana		*Gut microbiome*	*Tet(O)*, *tet(Q)*, *tet(W)*, *tet(X)*, *tetAB(P)*	N/R	[117]
	Guatemala		*Escherichia coli*	*Tet(A)*, *tet(B)*		[118]
	Panama				Tetracycline (30.0)	[119]
Asia	China		*Klebsiella pneumoniae*	*Tet(X)*	Tigecycline, tetracycline, doxycycline, minocycline, eravacycline, omadacycline (100.0)	[120]
	Taiwan	Clinical samples from patients	*Acinetobacter baumannii*		Tigecycline (N/R)	[121]
	Iran		*Escherichia coli*	*Tet(A)*, *tet(B)*, *tet(C)*, *tet(D)*	Tetracycline (60.0), doxycycline (44.0), and tigecycline (N/R)	[122]
	Korea			*Tet(A)*, *tet(B)*	N/R	[123]
	Armenia		*Klebsiella pneumoniae*	*Tet(A)*, *tetR(A)*, *tet(B)*, *tetR(B)*	Tetracycline (56.25) and tigecycline (6.25)	[124]
	Cambodia		*Escherichia coli*	*Tet(A)*	N/R	[125]
	India		*Klebsiella pneumoniae*	*Tet(A)*, *tet(B)*	Tigecycline (20.4)	[126]
	Iraq		*Staphylococcus aureus*	*Tet(K)*, *tet(38)*, *tet(L)*, *tet(M)*	N/R	[127]
	Saudi Arabia		*Group B Streptococcus*	*Tet(M)*, *tet(O)*, *tet(L)*	Tetracycline (76.5)	[128]
	Thailand		*Streptococcus suis*	*Tet(O)*	Tetracycline (98.2)	[129]
	Malaysia	Urine	*Escherichia coli*	*Tet(A)*, *tet(B)*	Tetracycline (51.7)	[130]
	Kuwait	Feces	*Enterobacteriaceae species*	*Tet(X)*	Tigecycline (0.8–4.1)	[131]
	Nepal			*Tet(32)*, *tet(W)*, *tet(40)*, *tet(Q)*, *tet(M)*, *tet(O)*	N/R	[132]
	Lebanon		*Escherichia coli*	*Tet(A)*, *tet(B)*, *tet(C)*	Tetracycline (42.0)	[133]
Australia and Oceania	Australia		*Campylobacter jejuni*	*Tet(O)*	Tetracycline (11.1)	[134]
			*Campylobacter coli*		Tetracycline (2.4)	
	New Zealand		*Shigella* spp.	*Tet(A)*, *tet(B)*	Tetracycline (33.3–100.0)	[135]
Africa	Egypt	Clinical samples from patients	*Klebsiella pneumoniae*	*Tet(A)*	Tetracycline (53.8)	[136]
	South Africa		*Klebsiella pneumoniae*, *Escherichia coli*, *Enterobacter* spp., *Citrobacter* spp.	*Tet(X)*	Tigecycline (7.2)	[137]
	Zambia		*Escherichia coli*	*Tet(A)*, *tet(B)*	Tetracycline (3.39)	[138]
	Nigeria	Feces		*Tet(A)*, *tet(B)*, *tet(M)*	Tetracycline (83.0)	[139]
	Cameroon			*Tet(A)*, *tet(B)*	Tetracycline (75.55)	[140]
	Ethiopia			*Tet(A)*, *tet(D)*	Tetracycline (92.0–100.0)	[141]
	Ghana			*Tet(A)*	Tetracycline (22.6)	[142]
	Kenya			*Tet(A)*, *tet(B)*	Tetracycline (50.0)	[143]
	Gambia		*Staphylococcus aureus*	*Tet(M)*, *tet(L)*, *tet(K)*, *tet(38)*	N/R	[144]
	Sudan		*Campylobacter jejuni*	*Tet(O)*	N/D	[145]
	Zimbabwe		*Bifidobacterium* spp.	*Tet(O)*, *tet(W)*	N/R	[146]
	Tanzania	Nasal cavity	*Escherichia coli*	*Tet(A)*, *tet(B)*	N/R	[147]

* N/D—not detected. ** N/R—not reported.

**Table 5:** Classification of tetracycline-class antibiotics under the AWaRe framework and the WHO lists of essential medicines.

Antibiotic	Generation	AWaRe Category	* WHO EML/EMLc Lists
Chlortetracycline	First	Watch	Topical use for trachoma, infectious keratitis, and infectious blepharitis
Oxytetracycline			
Tetracycline		Access	
Demeclocycline		Watch	Not listed
Penimepicycline			
Clomocycline			
Minocycline	Second	** Watch/Reserve	
Doxycycline		Access	Malaria, trachoma, cholera, pneumonia, ^ COPD
Lymecycline		Watch	Not listed
Rolitetracycline			
Metacycline			
Tigecycline	Third	Reserve	
Omadacycline			
Eravacycline			
Sarecycline		Watch	

* WHO EML/EMLc—World Health Organization Model List of Essential Medicines/Model List of Essential Medicines for Children. ** Oral/Intravenous. ^ COPD—chronic obstructive pulmonary disease.

**Table 6:** Tetracycline-class antibiotics in veterinary medicine according to the WOAH list of antimicrobial agents of veterinary importance.

Antibiotic	Species	* WOAH List
Chlortetracycline	Avian, bovine, caprine, equine, ovine, rabbit, swine	Critically important antimicrobials
Doxycycline	Avian, bovine, camel, caprine, equine, fish, ovine, rabbit, swine	
Oxytetracycline	Avian, bee, bovine, camel, caprine, equine, fish, ovine, rabbit, swine	
Tetracycline		

* WOAH—World Organization for Animal Health.

**Table 7:** Classification of tetracycline-class antibiotics according to the WHO list of medically important antimicrobials.

Antibiotic	Pharmacological Group	* WHO MIA List
Chlortetracycline	Tetracyclines	Highly important antimicrobials, authorized for both humans and animals
Oxytetracycline		
Tetracycline		
Demeclocycline		
Penimepicycline		
Clomocycline		
Minocycline		
Doxycycline		
Lymecycline		
Rolitetracycline		
Metacycline		
Sarecycline		
Omadacycline	Aminomethylcyclines	Authorized for use in humans only
Eravacycline	Fluorocyclines	
Tigecycline	Glycylcyclines	

* WHO MIA—World Health Organization List of Medically Important Antimicrobials.

**Table 8:** Advantages and disadvantages of tetracycline-degradation methods.

Degradation Method	Advantages	Disadvantages
Hydrolysis	Simple process, occurs naturally, no need for catalysts	Slow under neutral/low temperatures; produces bioactive intermediates with residual toxicity
Photodegradation	Fast under sunlight; can occur in surface waters; environmentally friendly	Limited to light-exposed areas; generates potentially more toxic intermediates
Oxidative degradation	Rapid degradation; applicable in wastewater treatment	Produces reactive byproducts with potential acute toxicity; sensitive to water chemistry
Biodegradation	Environmentally friendly; can mineralize tetracyclines to harmless products	Slow; incomplete degradation possible; some metabolites may retain bioactivity
Adsorption/Physical removal	Simple; immediate removal from water; can be combined with other methods	Does not destroy the compound; requires regeneration/disposal of adsorbent; does not prevent toxicity

## Data Availability

Not applicable.

## Supplementary Materials

The following supporting information can be downloaded at: https://www.mdpi.com/article/10.3390/antibiotics14121183/s1, Table S1: Global tetracycline consumption in human health over the past five years (2019–2025); Table S2: Global tetracycline consumption in animal health according to the ANIMUSE database.

## Abbreviations

The following abbreviations are used in this manuscript:

AMR	Antimicrobial resistance
AMS	Antimicrobial stewardship
ANIMUSE	Animal Antimicrobial Use
AWaRe	Access, Watch, Reserve
CDC	Centers for Disease Control and Prevention
DDD	Defined daily doses per 1000 individuals per day
EHL	Elimination half-life
EMA	European Medicines Agency
EML	Essential medicines’ Model List
EU	European Union
FAO	Food and Agriculture Organization
FDA	Food and Drug Administration
LMICs	Lower- and middle-income countries
LoD	Limit of detection
LoQ	Limit of quantification
MeSH	Medical Subject Headings
MIA	Medically Important Antimicrobial agents
MSC	Minimal selective concentration
NWQMC	National Water Quality Monitoring Council
PNEC	Predicted no-effect concentration
ROS	Reactive oxygen species
Tet	Tetracycline
UNEP	United Nations Environment Programme
U.S.	United States
VCIA	Veterinary Critically Important Antimicrobial agents
VHIA	Veterinary Highly Important Antimicrobial agents
VIA	Veterinary Important Antimicrobial agents
WHO	World Health Organization
WOAH	World Organization for Animal Health
WQP	Water Quality Portal
WWTPs	Wastewater treatment plants
