# Supplementary material for: Tetracyclines in the Modern Era: Global Consumption, Antimicrobial Resistance, Environmental Occurrence, and Degradation Techniques
Source: Antibiotics (Basel). 2025 Nov 21;14(12):1183. doi: 10.3390/antibiotics14121183 (PMC12729376; doi:10.3390/antibiotics14121183)
Supplement: Supplementary file 1 [file antibiotics-14-01183-s001.zip › Supplementary tables.pdf]

Table S1. Global tetracycline consumption in human health over the past five years (2019-2025).

World region	Country	Defined daily doses per 1,000 inhabitants per day	Year of data collection	Reference
European Union	Austria	0.4	2023	[33]
	Belgium	1.8		
	Bulgaria	2.5		
	Czechia	1.7		
	Croatia	1.1		
	Denmark	1.8		
	Estonia	1.6		
	Finland	2.7		
	France	2.8		
	Germany	1.6		
	Greece	1.95		
	Hungary	1.1		
	Ireland	4.0		
	Iceland	4.1		
	Italy	0.7		
	Lithuania	1.9		
	Luxemburg	1.7		
	Latvia	2.7		
	Malta	2.6		
	Netherlands	1.7		
	Norway	2.7		
	Poland	1.9		
	Portugal	1.0		
	Romania	0.9		
	Spain	1.5		
	Slovenia	0.6		
	Slovakia	1.8		
European Region of the World Health Organization	Turkmenistan	1.4	2022	[34]
	Montenegro	1.8		
	Serbia	1.8		
	Kyrgyzstan	1.0		
	Kazakhstan	0.6		
	Albania	2.6		
	Bosnia and Herzegovina	1.5		
	Georgia	0.5		
	Macedonia	0.3		
	Tajikistan	0.2		
	Belarus	1.3		
	Russia	0.9		
	Azerbaijan	1.4		

	Ukrain	0.8		
	Switzerland	1.2		
	Armenia	1.0		
Asia	Brunei Darussalam	1.0	2019	[35]
	Hong Long	1.8		
	Japan	0.9		
	Lao PDR	2.5		
	Malaysia	0.8		
	Mongolia	1.6		
	Philippines	0.1		
	Срштя	0.13		[36]
Americas	Argentina	0.9	2019-2022	[37]
	Barbados	4.2		
	Brazil	0.5		
	Chile	0.1		
	Colombia	1.5		
	Costa Rica	1.6		
	Guyana	0.9		
	Honduras	0.5		
	Paraguay	0.02		
	Peru	0.7		
	Saint Kitts	0.2		
	Nevis	0.2		
	Canada	1.2	2019	[38]
Africa	Ethiopia	1.47	2022	[40]
	Tanzania	17	2019	[39]

Table S2. Global tetracycline consumption in animal health according to the ANIMUSE database.

World region	Country	Milligrams per kilogram of estimated animal biomass	Year of data collection	Reference
Europe	Albania	1.85	2023	[44]
	Armenia	0.48		
	Azerbaijan	5.08		
	Belgium	5.27		
	Benin	5.32		
	Bolivia	0.96		
	Bosnia and Herzegovina	8.28		
	Bulgaria	15.00		
	Croatia	6.46		
	Cyprus	33.11		
	Denmark	2.93		
	Estonia	6.26		

	France	4.98
	Finland	1.47
	Georgia	8.50
	Iceland	0.57
	Ireland	6.45
	Italy	12.57
	Latvia	1.63
	Luxembourg	2.22
	Malta	11.48
	Moldova	26.63
	Montenegro	3.29
	North Macedonia	13.41
	Norway	0.03
	Portugal	23.75
	Romania	10.15
	San Marino	10.34
	Slovakia	2.68
	Slovenia	2.35
	Spain	17.70
	Sweden	0.45
	Switzerland	4.35
	Netherlands	6.12
	United Kingdom	5.39
Americas	Costa Rica	41.45
	Cuba	0.41
	Canada	39.24
	Costa Rica	41.45
	Saint Vincent and the Grenadines	2.64
Africa	Republic of the Congo	3.03
	Egypt	21.57
	Cabo Verde	0.16
	Eswatini (Swaziland)	3.65
	Kenya	5.74
	Gabon	9.33
	Mali	10.64
	Senegal	6.41
	Togo	2.31
Asia	Myanmar	10.81
	Sri Lanka	7.57
Australia and Oceania	New Caledonia	6.51
	New Zealand	0.88
